# Equity in prenatal healthcare services globally: an umbrella review

**DOI:** 10.1186/s12884-024-06388-0

**Published:** 2024-03-11

**Authors:** Zeenat Ladak, Nagma Grewal, Minji Olivia Kim, Stephanie Small, Alexia Leber, Mehdiya Hemani, Qiuyu Sun, Deena M. Hamza, Celia Laur, Noah M. Ivers, Olesya Falenchuk, Richard Volpe

**Affiliations:** 1 University of Toronto, Toronto, Canada; 2 University of Alberta, Edmonton, Canada; 3 McMaster University, Hamilton, Canada; 4 Women’s College Hospital Institute for Health System Solutions & Virtual Care, Toronto, Canada; 5 Women’s College Hospital, Toronto, Canada

**Keywords:** Prenatal, Antenatal, Pregnancy, Health services, Health equity, Inequity, Umbrella review, Review of reviews, PROGRESS-plus

## Abstract

**Background:**

Timely, appropriate, and equitable access to quality healthcare during pregnancy is proven to contribute to better health outcomes of birthing individuals and infants following birth. Equity is conceptualized as the absence of differences in healthcare access and quality among population groups. Healthcare policies are guides for front-line practices, and despite merits of contemporary policies striving to foster equitable healthcare, inequities persist. The purpose of this umbrella review is to identify prenatal healthcare practices, summarize how equities/inequities are reported in relation to patient experiences or health outcomes when accessing or using services, and collate equity reporting characteristics.

**Methods:**

For this umbrella review, six electronic databases were searched (Medline, EMBASE, APA PsychInfo, CINAHL, International Bibliography of the Social Sciences, and Cochrane Library). Included studies were extracted for publication and study characteristics, equity reporting, primary outcomes (prenatal care influenced by equity/inequity) and secondary outcomes (infant health influenced by equity/inequity during pregnancy). Data was analyzed deductively using the PROGRESS-Plus equity framework and by summative content analysis for equity reporting characteristics. The included articles were assessed for quality using the Risk of Bias Assessment Tool for Systematic Reviews.

**Results:**

The search identified 8065 articles and 236 underwent full-text screening. Of the 236, 68 systematic reviews were included with first authors representing 20 different countries. The population focus of included studies ranged across prenatal only (*n* = 14), perinatal (*n* = 25), maternal (*n* = 2), maternal and child (*n* = 19), and a general population (*n* = 8). Barriers to equity in prenatal care included travel and financial burden, culturally insensitive practices that deterred care engagement and continuity, and discriminatory behaviour that reduced care access and satisfaction. Facilitators to achieve equity included innovations such as community health workers, home visitation programs, conditional cash transfer programs, virtual care, and cross-cultural training, to enhance patient experiences and increase their access to, and use of health services. There was overlap across PROGRESS-Plus factors.

**Conclusions:**

This umbrella review collated inequities present in prenatal healthcare services, globally. Further, this synthesis contributes to future solution and action-oriented research and practice by assembling evidence-informed opportunities, innovations, and approaches that may foster equitable prenatal health services to all members of diverse communities.

**Supplementary Information:**

The online version contains supplementary material available at 10.1186/s12884-024-06388-0.

## Introduction

Timely, quality healthcare should be available and accessible to all individuals, and the policies that guide healthcare decision making should foster equitable care. However, globally, achieving this has proved to be challenging [1–3]. Broadly, equity is conceptualized as the absence of differences in healthcare access and use among population groups, and that all population groups can achieve the health outcomes of the most socially advantaged [4, 5]. Prominently, evidence suggests that healthcare inequities disproportionately affect women’s, maternal, birthing individuals’ health, infant development, and family wellbeing [3, 6].

Globally, major health organizations have categorized prenatal health as encompassing overall maternal health during pregnancy [3, 7]. The prenatal period is defined as the time from conception of pregnancy up to delivery. Evidence suggests that suboptimal health outcomes during this life-stage stem from inequitable access to and subsequent engagement in prenatal care services [8, 9]. Studies have identified that inadequate prenatal care can result in a higher risk of complications during and after pregnancy for the birthing individual and infant [10–16]. Our review focuses on the prenatal period as adequacy of care during this time can influence subsequent physiological and psychological experiences during birth and the postpartum period [10–17]. Inequities are rooted in systemic factors such as institutional racism, and social and economic inequities that influence one’s social determinants of health [18]. Common patient reported challenges include geographical proximity (e.g., rural and remote settings), communication barriers, financial barriers, lack of cultural safety, and a lack of known services. These challenges have been exacerbated during the COVID-19 pandemic [1, 3, 6].

Despite the merit of contemporary policies that strive to foster the conditions for health equity for all, inequities in maternal healthcare persist. For example, inequities can be observed in access to services such as consultation with a healthcare professional (i.e., general practitioner, obstetrician, gynecologist, midwife), timely prenatal screening, and prevention and early intervention for maternal mental health needs [1, 3]. Complicating matters further, evidence suggests that individual practitioners’ interpretations of policies may contribute to variability in application of these guidelines resulting in inconsistent implementation of everyday practices with diverse populations [19, 20]. As such, research aimed at addressing birthing individuals’ access to and use of prenatal healthcare is necessary at practice and policy levels, to ensure that care is both equitable and effective in improving the health of prenatal patients [1, 3, 21]. Prenatal healthcare services include care provided to a birthing individual, to prevent complications of pregnancy and to ensure the wellbeing of the birthing individual and infant following birth [17, 22, 23]. Examples of these services include, but are not limited to, visiting a healthcare professional or community health worker in person or through virtual care for a physical exam, a fetal ultrasound, prenatal genetic testing or screening, gestational diabetes screening, birth planning, nutrition, substance use and mental health consults [22, 23].

The aim of this umbrella review was to identify and summarize practices within prenatal healthcare services as they relate to equity/inequity and explore barriers and facilitators of how equities/inequities influence the patient experience or health outcomes when accessing/using services, and to review how equity is reported. We intended to identify both qualitative and quantitative systematic reviews that investigated primary studies for practices in prenatal healthcare. This review provides an overarching scan of existing evidence of prenatal healthcare practices globally and a platform to critically discuss their contribution to reduce inequity present in prenatal healthcare, and plausible solutions to improve equity.

## Methods

### Umbrella review methodology

There has been an influx of systematic reviews on the topic of equity influencing access and use of prenatal care. It is becoming increasingly difficult for healthcare professionals, policy makers, and researchers to review the volume of evidence-generating literature to guide evidence-informed actions. An umbrella review (also termed “overview of reviews” or “review of reviews”) consolidates the content captured in systematic reviews and meta-analyses [24–28]. The umbrella review provides a solution by packaging mass information into a synthesized and focused document for decision-makers, including healthcare professionals and policy makers, to efficiently incorporate evidence into their own contexts [24, 26–28]. Further, the umbrella review methodology allows us to capture the way in which equity is conceptualized and reported in the included studies [29–31]. There is still much variability in how equity is reported in systematic reviews; we used the recommended Campbell and Cochrane Collaboration’s PRISMA-Equity extension (Supplementary file 1) as a guide to encourage more standardized data extraction and reporting [31]. To ensure a thorough review of equity factors, we also used the PROGRESS-Plus equity framework to guide this work as it offers a comprehensive set of factors to consider as potential sources of inequity population-wide, and it is meant to complement the PRISMA-Equity extension [31, 32].

### Protocol

The protocol for this umbrella review was registered with PROSPERO (CRD42022301574) [33]. Any changes to the protocol were documented and can be viewed online.

### Eligibility criteria

The eligibility criteria for this review followed the PICOS (population, intervention, comparison, outcome, and study design) framework. Detailed inclusion and exclusion criteria are listed in Table 1. A significant aspect of our inclusion criteria was that outcomes were required to include an explanation of *how* equity/inequity influenced prenatal patient experience or health outcomes; a factual/statistical relation/association was not sufficient to be included in this review. The rationale for this was the need to develop a greater understanding of the mechanisms involved that lead to equity/inequity in different contexts and how decision-makers can adapt them to improve health equity in prenatal care.

**Table 1:** Inclusion and exclusion criteria for study screening and selection

PICOS	Inclusion Criteria	Exclusion Criteria
**Population**	- Must contain a prenatal human patient population at any stage during pregnancy from conception (0 days gestation) up to delivery - May include gender identities other than women	- Non-human prenatal patients (i.e., animal models, cell models) - Focused only on postnatal or non-pregnant human patients
**Intervention**	- Any healthcare service or practice where the patient interacts with the healthcare system, that includes an outcome in relation to the target population health or healthcare experience	- Healthcare service or practice related only to contraceptives, abortion, ectopic pregnancies, and fertility - Healthcare service or practice that does not include outcomes related to target population (i.e., if it focuses on healthcare professionals only, or focused on a procedure/method and not the patient outcome) - Focus on policy or guidelines rather than healthcare service or practice interaction
**Comparison**	- An alternate intervention within prenatal healthcare, or a control for no intervention, or an internal comparison of outcomes	- None
**Outcome**	- Pregnancy outcomes or experience of prenatal care based on explaining how care was influenced by equity/inequity	- No prenatal population outcomes or experience - Prenatal population outcomes or experience which only stated a factual/statistical relation/association to equity/inequity
**Study Design**	- Studies identified as systematic reviews or meta-analyses if they included a systematic search strategy with two or more databases, a clear inclusion criterion, and a focus on primary research studies - Published in English language - Studies must mention “equity”, “inequity”, “equitable”, or “inequitable” in their title, abstract, introduction, methods, results, discussion, or conclusion. - Published in any region or country	- Studies which are not systematic reviews - Any other review type (i.e., umbrella, scoping, narrative, integrative, critical, literature review) - Any primary studies - If “equity”, “inequity”, “equitable”, or “inequitable” is not mentioned in the body of the article

### Search strategy and selection

A systematic search strategy was developed with support from two librarians (EM, JW) and was used to retrieve relevant systematic reviews and meta-analyses [34, 35] from six electronic databases (Ovid: Medline, Embase, APA PsycInfo; EBSCO: CINAHL; ProQuest: International Bibliography of the Social Sciences; Cochrane Database of Systematic Reviews). Search terms included: *prenatal, antenatal, prepartum, pregnancy, equity,* and *inequity*; truncations and variations were used where relevant. No limitations on date were used during the search. Hand searching of reference lists was also completed for all studies included in the review to ensure any systematic reviews that may have been missed from the systematic search, were included. The complete search strategy is available in Supplementary file 2. The original search was performed in January 2022 and updated in August 2022.

Covidence is a web-based collaboration software platform that streamlines the production of systematic and other literature reviews [36] and was used to organize and carry out the screening process accurately. All identified articles were uploaded to Covidence and duplicates were removed. Title, abstract, and full text screening were completed independently, in duplicate, by two reviewers (ZL, NG, MOK, SS, AL, MH, QS); the second reviewer was always the lead author (ZL). Any conflicts were resolved through discussion. Tracking of included articles and reasons for excluded articles was done through Covidence and later recorded manually using a Microsoft Excel spreadsheet.

### Data extraction, analysis, and synthesis

A Microsoft Excel spreadsheet was used for data extraction and was designed through an iterative process, and included revisions between two authors (ZL, NG). To pilot the data extraction sheet, three full text articles were extracted independently, in duplicate, by two authors (ZL, NG). Any conflicts were resolved through discussion and the data extraction sheet was adjusted and optimized as appropriate. After the data extraction sheet was finalized, each full text article was extracted independently by one reviewer (NG, SS, MH, AL, MOK, QS), and all extractions were reviewed for accuracy by the lead author (ZL).

Extracted data included publication characteristics (author, country, year of publication, journal, funding source, title), study characteristics (research question, population, intervention, comparators, study designs), equity reporting (definition, when and where equity is mentioned, equity related frameworks), primary outcomes, and secondary outcomes. The primary outcome included how an equity factor influenced the prenatal patient experience or health outcome, while the secondary outcome included how equity/inequity during pregnancy impacted subsequent infant/child health and development.

The primary and secondary outcomes were deductively analyzed and mapped to the PROGRESS-Plus framework as barriers and facilitators to equity in health services. PROGRESS refers to place of residence, race/ethnicity/culture/language, occupation, gender/sex, religion, education, socioeconomic status, and social capital. Plus refers to other equity factors not listed such as age [32]. Inductive themes were also generated. Deductive and inductive themes are illustrated through maximum variation to capture themes within and across the studies [37].

The primary objective to review equity reporting characteristics was analyzed by summative content analysis techniques [38, 39]. The terms ‘equity’ and ‘inequity’ and their truncated equivalents (equit*, inequit*) were searched and counted in each included systematic review. Counts were analyzed using descriptive statistics in Microsoft Excel. Additionally, any equity/inequity definitions were sought and compared.

### Quality assessment

Each included systematic review was assessed for quality by the lead author (ZL) using the Risk of Bias Assessment Tool for Systematic Reviews (ROBIS), which evaluates the risk of bias within systematic reviews [40]. The ROBIS includes four domains and a final overall review of risk of bias. The scoring of each domain was categorized into low, moderate, and high risk of bias. Specific scoring categories and scores for each included study can be seen in Supplementary file 3.

## Results

**Fig. 1:**
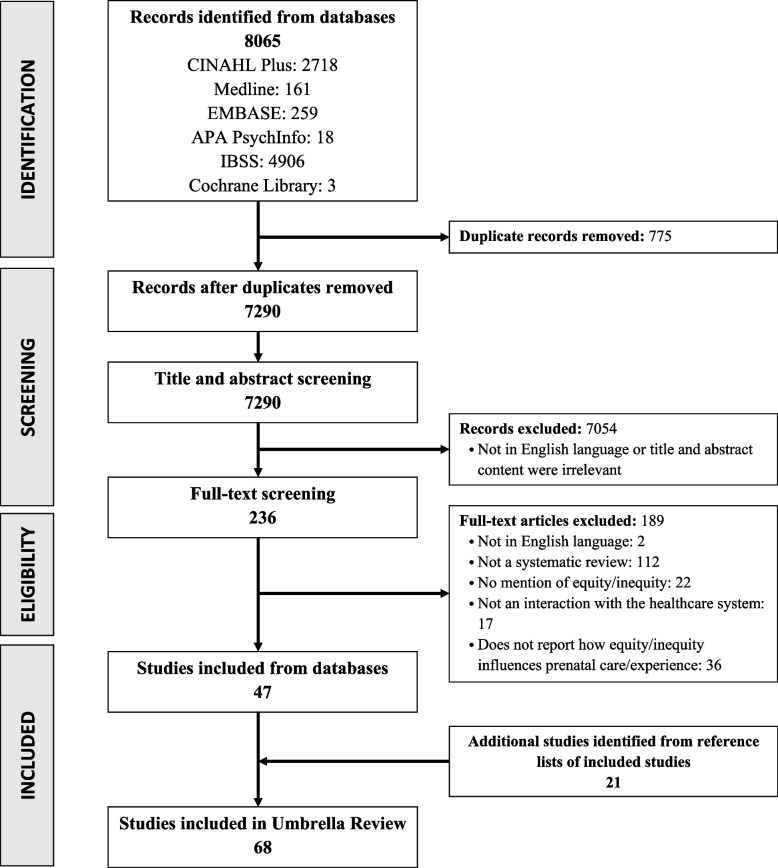
PRISMA flow diagram of literature search and selection process

The systematic search captured 8065 articles (Fig. 1). Of the 236 articles reviewed during secondary, full-text screening, 68 systematic reviews were included in this umbrella review (Fig. 1). A condensed summary of study characteristics and outcomes for each included review can be viewed in Supplementary file 4 and all excluded studies with reason for exclusion can be viewed in Supplementary file 5. To address the aims of this review, we present findings in six categories: Study Characteristics; Study Foci, to identify and summarize practices within prenatal healthcare services; Impact of Equity/Inequity on Prenatal Care and Other Factors Impacting Access/Use of Prenatal Care, to explore barriers and facilitators of how equities/inequities influence the patient experience or health outcomes when accessing/using services; Equity Reporting Characteristics to review how equity is reported; and Quality Assessment.

### Study characteristics

**Fig. 2:**
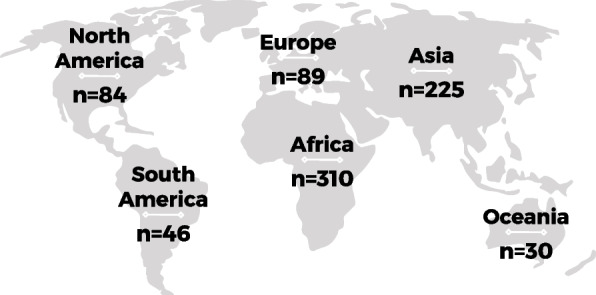
Global distribution of studies within included systematic reviews. Values represent the number of studies within the included systematic reviews that were published within the labeled continent. Studies not reported: Malqvist 2012, Jhaveri 2021

**Fig. 3:**
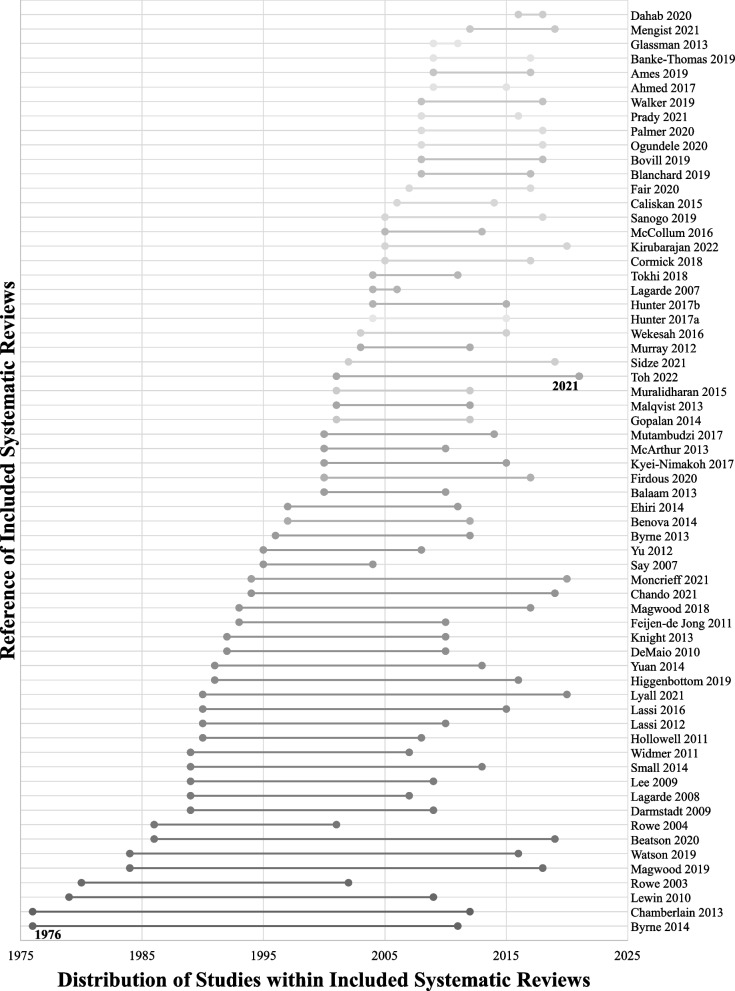
Publication timeline distribution of studies within included systematic reviews. Horizontal lines represent the publication year range from earliest to latest of studies within the included systematic reviews. Studies not reported: Malqvist 2012, Victoria 2012, Vanstone 2019, Jhaveri 2021

Of the 68 included systematic reviews, 13 were meta-analyses. The methodology of included studies within the reviews varied; 33 included mixed methods studies, 23 included quantitative studies only, 10 included qualitative studies only, and two reviews did not report their methods clearly. The majority of study first authors were from the United Kingdom (*n* = 23), Australia (*n* = 18), Canada (*n* = 7), and United States of America (*n* = 6). The studies analyzed within the included reviews were distributed across the globe (Fig. 2, Supplemental file 4), with the largest proportion of studies from Africa (*n* = 310), followed by Asia (*n* = 225). Studies from Oceania (*n* = 30) and South America (*n* = 46) were the least represented. All included systematic reviews were published in or after 2003, with 15 published during or after the year 2020. Of these, four included analyses of studies during or after the year 2020 [41–44] (Fig. 3). Figure 3 also shows the distribution across time of the publication year of all studies within the included reviews, with the earliest being in 1976 and the most recent in 2021.

### Study foci

To identify and summarize practices within prenatal healthcare services, we reviewed the population and health service topic of focus for each included study. The included systematic reviews focused on various populations including prenatal only (*n* = 14), perinatal (*n* = 25), maternal (*n* = 2), maternal and child (*n* = 19), and a general unspecified population (*n* = 8). The reviews also focused on a range of topics including prenatal healthcare services such as prenatal testing/screening, smoking cessation, mobile-health (mhealth)/virtual health, lay/community health workers (CHWs), and mental health, and other services associated with prenatal healthcare such as conditional cash transfer (CCT, i.e., income subsidies) and faith-based/community organizations. A condensed summary of healthcare services for each included review can be viewed in Supplementary file 4.

### Impact of equity/inequity on prenatal care

The included systematic reviews provided insight on how barriers and facilitators of equity/inequity in prenatal healthcare impacts the patient experience or health outcomes when accessing/using care, and subsequently infant and child health. Much of the data overlaps across PROGRESS-Plus factors, however, Tables 2 and 3 summarize the data into each of the factors. A condensed summary of equity related outcomes for each included review can be viewed in Supplementary file 4.

**Table 2:** Barriers and facilitators of equity in prenatal healthcare practices that impact access or use of health services

PROGRESS-Plus Factor	Barriers and Facilitators of Equity
**Place of Residence**	**Barriers** Despite availability of innovations (i.e., CCT, home visitation, CHWs), remote/rural patients face difficulties in accessing or having knowledge of resources because of geographical remoteness and poor transportation options, especially during obstetrical emergencies [41, 42, 45–52]. **Facilitators** Availability of multipurpose healthcare professionals facilitates provision of prenatal care in remote areas [53]. Community referrals, CHWs, CCTs, and transport innovations reduce referral times, improve access to care for rural/remote patients, and reduce adverse outcomes [54–57]. CCTs that distribute resources directly to communities avoid transportation barriers [58]. Telemedicine innovations increase access and use of care, and improves patient satisfaction [56, 59, 60]. Home visiting programs reduce transport/mobility/communication barriers, improve access to care, and improve pregnancy outcomes [46, 61–64].
**Race, Ethnicity, Culture, Language, Religion**	**Barriers** Non-White/European patients are less likely to initiate, book late or fewer prenatal appointments [65–68] and show lower uptake of prenatal testing and screening because they are less likely to be offered the service or provided with information or consent compared to White/European individuals [49, 65, 66, 68]. Non-White/European patients experience greater unfair, discriminatory treatment compared to White individuals which leads to a greater risk of adverse birth outcomes [69, 70]. Cultural (i.e., smoking as a spiritual practice) or religious (i.e., not have other people examine one’s body) norms and perceptions of distrust and patriarchy in the western healthcare system and lack of healthcare professionals with similar ethnic or cultural background leads to delayed initiation of prenatal care by patients and feelings of being unwelcome, patronized, and an unsafe pregnancy [43, 44, 49–51, 53, 58, 68, 70–76]. Cultural norms of family members making decisions on behalf of the patient leads to uninformed decisions [50, 68, 77]. Patients that spoke the language or who were born in the country have a greater knowledge of healthcare practices and access to care [68, 70, 74, 78] compared to those with communication difficulties, especially without adequate interpretation services [70, 73, 75–77]. Language barriers, lack of cultural appreciation, poor attitudes, and reluctance among healthcare professionals limits opportunities of religious and ethnic minority patients and leads to these patients feeling unsupported, devalued, and fearful [43, 49, 50, 52, 68, 74–76, 79]. Patients who are immigrants and ethnic minorities experience a lack of communication and receive inadequate access to services. Some even avoid maternal healthcare because they perceive or actually receive a different quality of care and health education or they want to prevent being discriminated against by healthcare professionals [44, 49, 50, 52, 53, 58, 70, 71, 73, 74, 76, 80]. Programs targeted at lower socioeconomic groups do not effectively reach ethnic minority patients, as such these populations receive incomplete benefits [79]. **Facilitators** CHWs (including Aboriginal Health Workers) improve health education, increase prenatal care attendance, reduces stress, and increase healthy habits (i.e., smoking abstinence) for non-White and Aboriginal patients, and those of non-Western culture [46, 55]. Maternity care services (e.g., midwifery) adapted to patient′s expectations enhance the patient experience by reducing anxiety, creating a sense of cultural safety, and allowing patients to feel valued and to take control of their pregnancy. Examples of adaptations include interpretation services, social support, cultural knowledge, cross-cultural training of healthcare professionals, and relevant and easy to understand information [42–44, 49, 51, 71, 74, 75]. Virtual health innovations that incorporate local language use improve access to care and ease of use [81].
**Occupation**	**Barriers** Unemployed patients and their partners book late or fewer prenatal appointments and employed face barriers in taking time off work due to financial constraints or for family obligations [50, 65, 75, 77, 79].
**Gender/Sex**	**Barriers** Gender norms (i.e., women cannot travel alone, make decisions, or they must stay home to take care of their children) lead to delayed care and underuse of health services proportionate to needs and feelings of powerlessness and loss of autonomy [49, 58, 62, 70, 71, 74, 77, 79, 82]. Lack of available female staff leads to patients delayed seeking of care or feelings of embarrassment [44, 49, 77, 79, 82]. Lack of a female support system leads to patients feeling less confident to discuss their concerns with healthcare professionals [71]. Experiences for LGBTQ2S+ identifying patients are distressing because of the frequency of use of sex-specific words, assumptions that patients are women, lack of healthcare professionals’ knowledge or acknowledgement [42]. **Facilitators** Targeted gender innovations that encourage men to support women, promote women’s autonomy, and provide health education, increase care use, improve nutrition, improve mental health, and reduce adverse pregnancy outcomes [49, 52, 77, 83]. Home visitation programs are valuable to provide health education and care to women who were disadvantaged by gender norms [46]. Strategies focused on using gender-neutral pronouns, inclusive tools, and trauma-informed training for healthcare professionals improve experience and enhance comfort for LGBTQ2S+ identifying patients [42].
**Education**	**Barriers** Lower levels of patient and partner education are associated with lack of health education [79] and leads to a delayed initiation of or infrequent use of prenatal care by patients and increased risk of adverse pregnancy outcomes [53, 62, 70, 77, 79, 82, 84]. Patients with a higher level of education tend to have greater authority during their pregnancy [82]. Despite available innovations, patients are unaware of their eligibility, lack knowledge of services or lack general health education and therefore do not seek services which can lead to greater pregnancy complications or maternal near miss situations [58, 64, 85]. When services are utilized, some patients are still provided with misinformation [43, 44, 64]. **Facilitators** CHWs (including Aboriginal Health Workers), birth preparedness, and home visitation programs improve patient’s health education (including smoking cessation), confidence and preparedness, and care-seeking habits which leads to less maternal stress and prevention of obstetrical complications and improves nutritional status [46, 47, 55, 59, 63, 86–89]. Home-based records target and improve patient and family knowledge and lead to improved confidence and sense of empowerment, increases prenatal care attendance, and improved recognition of pregnancy complications [90, 91]. Media campaigns and health education programs increase patient knowledge, awareness and readiness during pregnancy and lead to improved health outcomes [88, 92, 93].
**Socioeconomic Status**	**Barriers** Patients of lower SES show lower receipt and uptake of prenatal care [50, 74, 94–96], testing, and screening [41, 66] because of barriers accessing care and stigmatizing behaviour they receive [70]. Many patients also worry about loss of income and care seeking costs and therefore work right up to delivery [79]. Nutrition supplementation (e.g., iron, folic acid) coverage favours the wealthiest over poorest patient households and leads to a greater proportion of anemic patients of poor households [94]. Increased fees for care limits access for patients [53, 72], fees external to innovation/service costs (i.e., nicotine therapy), and narrow eligibility of innovations (i.e., CCT) also prohibit patients from using services [42, 45, 46, 50, 52, 58, 64, 75, 96–98]. Patients of low SES households have limited access to phones, cellular or internet networks or electricity and therefore cannot engage in virtual health innovations [78]. **Facilitators** Free/universal healthcare, reducing user fees, public assistance and insurance programs, or CCT innovations leads to increased household income/spending. This increases access to and use of services for socioeconomically disadvantaged patients and improves health education [45, 53, 54, 56, 57, 59, 62, 67, 92, 99–105], nutritional status [99], and pregnancy outcomes [45, 59, 61, 62, 101, 102], reduces pregnancy complications, and develops a sense of empowerment for patients [45]. CCTs that include cost coverage that may be indirectly associated with care services (i.e., travel) improves access to care [58]. Targeted nutrition programs improve knowledge and practices of dietary habits and supplements during pregnancy among the poorest households [94]. Innovations such as CHWs that actively connect prenatal patients of low/middle-income with care during pregnancy including home visits, increases service utilization and timeliness, improves preparedness, and reduces adverse outcomes [54]. CHWs improve health education and reduce smoking, for low-income patients [55].
**Social Capital**	**Barriers** Personal/family priorities including childcare may conflict with available innovations, especially with lack of family support, and those who are socially excluded face barriers including lack of knowledge [45, 50, 51, 75]. Nepotism and personal connections influence availability of services [46]. **Facilitators** Faith-based and community organizations provide higher-quality care, increased referrals, greater access to services, improve health education, pregnancy outcomes, and prenatal attendance [56, 59, 103, 106]. Additionally, family involvement has an even greater impact on these outcomes [107]. Innovations that encourage significant relationships, family and partner support, peer support, and community support, positively influence the patient’s well-being and health habits (e.g., smoking abstinence) and their relationship with their baby [49, 51, 59, 64, 71, 83, 89, 93, 108]. Tailored psychosocial support innovations co-developed or led by patients improve pregnancy and birth outcomes, improve cultural appropriateness, and are valued by patients [51, 59, 63, 64, 75, 88, 108]. Virtual health innovations that incorporate an interactive online community improves interaction between patients and with healthcare professionals [60, 78, 81].
**Plus—Age**	**Barriers** Patients of older age or that have previous experience with pregnancy have greater authority during their pregnancy [82], however, young and older women are still treated biased in terms of care quality [70]. **Facilitators** Mobile and electronic health innovations improve retention of patients under 18 years [59].

*CCT* conditional cash transfer, *CHW* community health worker, *SES* socioeconomic status

**Table 3:** Impact of equity/inequity in prenatal healthcare on infant health or development

PROGRESS-Plus Factor (s)	Impact of Equity/Inequity
**Place of Residence**	CHWs provided community options of service and transportation, reducing adverse infant outcomes [55, 56].
**Race, Ethnicity, Culture, Language, Religion**	Tailoring interventions to local traditions and customs led to lower adverse infant outcomes [54].
**Gender/Sex**	Targeted gender innovations that encouraged men to support women and provide health education increased fathers’ knowledge of newborn care, early breastfeeding, and improved child nutrition and health outcomes [59, 83].
**Education**	Lack of or misleading health education led to a delayed initiation of prenatal care by patients and increased risk of adverse outcomes for newborns [64, 74, 79]. CHWs and home visit programs improved patient’s health education, birth and post-partum preparedness, newborn care practices and care-seeking habits which led to reduced adverse infant outcomes [63, 86, 89]. Home-based records allowed for health education and knowledge to facilitate care continuity, improved newborn health outcomes, and increased paternal involvement in childcare [90, 91].
**Socioeconomic Status**	Birthing individuals of low-income families exposed to CCTs or reduced user fees during pregnancy led to increased use of infant/child health services and improved newborn outcomes and health [45, 97, 102, 105] including improved nutrition, reduced stunting and underweight, and increased use of health services compared to birthing individuals that did not receive CCTs [57, 99, 101]. Targeted nutrition programs improved knowledge and behaviour change of caregivers which led to increased growth and reduced anemia in poorest infants [94]. Free healthcare or reduced user fees increased access to and use of services for children [100] and family insurance coverage led to reduced adverse infant outcomes [61].
**Social Capital**	Faith-based and community organizations improved newborn outcomes and increased early breastfeeding [56, 106]; and family involvement had an even greater impact [107]. Psychosocial support services improved birth danger sign recognition and newborn care [59].

*CCT* conditional cash transfer, *CHW* community health worker, *SES* socioeconomic status

#### Place of residence

Transportation was a challenge for individuals living in remote or rural areas globally leading to a lack of access and use of services and a greater chance of adverse pregnancy outcomes (e.g., maternal mortality and morbidities, preterm birth, low birth weight, stillbirth), especially during emergencies. However, there were many facilitators with the potential to reduce this challenge, such as resources brought directly to communities and patients, including CHWs and home visiting programs. Virtual care was a facilitator identified commonly in Asian and African countries along with CCT strategies which were useful in reducing transport fees. The mention of multipurpose healthcare professionals was only identified in one study which analyzed settings across Asia, Africa, and South America (Table 2) [53]. The positive impact of CHWs also extended to infant health as utilization of services increased because of the reduced transportation barrier (Table 3).

#### Race, ethnicity, culture, language and religion

For the purpose of this review, we combined two PROGRESS-Plus factors, Race/Ethnicity/Culture/Language and Religion as most of the relevant data was associated with all or most of these factors. There was evidence of prenatal patients encountering discrimination when accessing and receiving care, poor service and care quality if offered at all, stigmatizing behaviour, and a lack of cultural appreciation, which led to a greater risk of adverse outcomes and lower utilization of care. These experiences were mostly associated with those in North America, Europe, and Oceania, who were non-White/European, immigrants, unfamiliar with the common language or western medicine culture, and of minority religions. Much of the data speaks to patients feeling unsupported, devalued, and even fearful, and leads them to avoid accessing care all together. Globally, studies identified facilitators to achieving equity. This included CHWs to improve health education among minority individuals and virtual innovations to incorporate local languages. Many studies mentioned adapting healthcare services to meet patient expectations by incorporating cross-cultural training to reduce patient anxiety and increase a sense of cultural safety (Table 2). This adaptation and tailoring of innovations also reduced the incidence of adverse infant or child outcomes (e.g., neonatal mortality, neonatal morbidities, stunting) in Asian, African, and South American countries (Table 3).

#### Occupation

The review captured information from South America, Asia, and Africa about unemployed patients and their partners booking late or fewer prenatal appointments, while employed individuals faced barriers in taking time off work due to financial constraints or for family obligations (Table 2). There were no facilitators of equity identified from the data.

#### Gender/sex

Gender norms globally (i.e., women cannot travel alone, cannot make decisions, or they must stay home to take care of their children) contributed to delays or underuse of care because of powerlessness in decision-making processes. The underrepresentation of women in both healthcare (i.e., staff or healthcare professionals) and personal support systems (i.e., peers or family members) was found to deter some individuals from accessing prenatal health services across continents. Additionally, the lack of healthcare professionals’ knowledge or inclusivity of LGBTQ2S+ groups led to distressing experiences for patients in North America, Europe, and Oceania (Table 2). To overcome these barriers, studies explained the use of innovations that encouraged men and partners to support and promote the birthing individual’s autonomy which improved health education, care use, reduced adverse outcomes of pregnancy and infant health, improved newborn care, and improved maternal and infant nutrition (Tables 2 and 3). Home visitation programs in Asia, Africa, and South America were also useful in providing information to women who were disadvantaged by gender norms. In North America, Europe, and Oceania, the use of inclusive strategies (e.g., the use of gender-neutral pronouns) was mentioned to support LGBTQ2S+ patients in feeling comfortable and improve the patient experience (Table 2).

#### Education

Lower levels of patient or partner education were associated with a lack of health education and led to delayed initiation or reduced use of care across the globe. A lack of health education was reported frequently as a cause for underutilization and adverse pregnancy outcomes (Table 2). Additionally, even with health education it was common for misinformation to be provided to patients, which increased the risk of adverse outcomes for patients and their newborns (Tables 2 and 3). CHWs, birth preparedness, and home visitation programs have been used to improve patient education and self-confidence which prevented adverse outcomes. Home-based records are paper or electronic documents that pregnant women and caregivers can use in the household to monitor their health and the health of their children [90]. Home-based records have also been implemented to improve health education and readiness during pregnancy and for newborn care (Tables 2 and 3).

#### Socioeconomic Status (SES)

Patients of low SES across the globe reported a reduced uptake of prenatal care because of stress surrounding loss of income, cost of services, and experiencing stigmatizing behaviour from healthcare professionals (Table 2). Innovations that overcame these barriers, including CCTs, reducing user fees, or public assistance programs, led to increased use of services by patients with strained financial status and improved health education, health outcomes during pregnancy, and health outcomes for newborns (Tables 2 and 3). These innovations also empowered patients to seek care in Asian and African settings. CHWs in South America, Asia, and Africa assisted by actively connecting patients of low SES to care during pregnancy (Table 2). Despite availability of innovations including CCT and CHWs, financially secure populations were prioritized over populations with lower SES, but the motivation and rationale for this was not included in the reviews (Table 2).

#### Social capital

Social capital barriers across the globe included personal/family priorities and lack of family support that may conflict with accessing care. Not knowing a health professional directly or limited personal networks were reported as factors leading to reduced opportunities to access prenatal healthcare services in Asia, Africa, and South America (Table 2). In similar settings, faith-based and community organizations have been successful in improving access to care for those that may be socially reserved or excluded; they increased referrals, improved prenatal attendance, and improved health outcomes (Tables 2 and 3). These organizations were more successful when families were involved. In general, the findings indicate that in-person or virtual innovations encouraging significant relationships and psychosocial support improved pregnancy and infant health outcomes (Tables 2 and 3).

#### Age (Plus)

Patients older or younger than the average reproductive age (i.e., 15–49 years) had different experiences during pregnancy in Asia and Europe. In some Asian cultures, older age was associated with greater authority if patients had previous experience with pregnancy, while younger aged patients received biased treatment. In Africa, Electronic health innovations (e.g., virtual health, mobile innovations) have been helpful in facilitating patient retention for those that were under 18 years of age (Table 2).

### Other Factors Impacting Access/Use of Prenatal Care

Across the PROGRESS-Plus factors, this study identified integrated themes that impacted access and use of prenatal care, that may or may not have been influenced by equity. Integrated themes include adequate prenatal care, patient-centred care, team-based care, continuity of care, multiple innovations, privacy and confidentiality, healthcare professionals’ assumptions, health system challenges, and care not benefiting the most in need when interventions are spread and scaled. The concept of adequate or inadequate prenatal care was mentioned in included studies which spanned analysis across all continents [45, 67, 71, 104], but definitions of ‘adequate’ varied or were not defined at all. Patient-centred care globally took the form of healthcare professionals’ attitudes, behaviours, and targeted care. All of which influenced whether patients would seek care or be satisfied with the care they received [42–46, 49, 51, 52, 60, 61, 71–74, 77–79, 81, 82, 88, 89, 91, 108]. Team-based or interprofessional care was a common theme across studies that included North American, European, and Australian settings; many explained how shared care increased quality and use of services and enhanced comprehensive care for patients [61, 67]. Continuity of care was also a recurring theme across continents, predominantly in North American, European, and Australian settings; it was important for patients to know that their healthcare professionals understood their journey, which further built a meaningful relationship [43, 49, 73, 74]. The approach of using multiple interventions to achieve equity was successful in studies across the globe, with an emphasis in African, Asian, and South American contexts, to ensure that patients received support from different avenues, as equity is complex and it is likely that more than one factor influenced their care [56, 59, 62, 63, 103, 108]. Privacy and confidentiality were also brought up as concerns in the data, specifically for electronic health innovations. Patients were uncertain of how their health data was stored and used; this was also influenced by technological literacy and was identified in studies that included countries across all continents [78, 91].

Stereotyped inequities of patients related to culture, religion, or ethnicity were included as barriers related to healthcare professionals’ assumptions, which led to unfair treatment in the United Kingdom. Examples of assumptions included that Muslim individuals did not want prenatal care or some cultures would be against terminating an affected pregnancy and hence these populations were less likely to be offered services including prenatal screening [66]. Studies with analyses predominantly in South America, Asia, and Africa acknowledged healthcare system challenges that increased opportunity for inequity including capacity burdens of health facilities and overworked healthcare professionals that led to deterioration in service quality [47, 49, 50, 52, 54, 72, 82, 99]. A consistent theme across the data, and most common in studies that included Asian and African countries, was the notion that interventions that attempted to overcome health inequities were not effective in reaching marginalized populations. Within this theme, studies suggested an increased need to explore implementation and evaluation characteristics to uncover how to better target innovations in different contexts, for patients with different circumstances, to ensure successful spread and scale and to avoid further contribution to equity gaps [47, 54, 60, 75, 90, 92–94, 96, 97, 101].

### Equity reporting characteristics

Equity reporting characteristics of the included reviews were assessed based on the use and frequency of the term equity or inequity, or truncated equivalents (Table 4). On average, included reviews mentioned the terms equity or inequity 11.9 times in their articles, with 120 being the greatest and one being the least frequent, which depicts the variation in the significance of the use of the terms. The mode presented as two mentions of the terms across all included studies. Only seven of the studies included equity/inequity as part of their article title, and 36 included it in their abstracts. The majority of equity/inequity counts were identified in the discussion section of the papers. When exploring the use of the terms in the entirety of the reviews, 19 articles mentioned equity/inequity only in the introduction, discussion, and/or conclusion sections, while only nine used the term in all sections. We also explored whether reviews defined equity/inequity or health equity/inequity and only five of the 68 included reviews provided definitions [46, 47, 79, 89, 94]. From the included studies, 51 were published on or after 2013, and of these, only three used the PRISMA-Equity 2012 checklist to guide their review [46, 47, 70]. Other frameworks related to equity that were a part of the reviews included PROGRESS-Plus [46, 47, 103, 109], an Indigenous Māori analytical framework [64], the Access to Care Framework [82], and the Stigma Action Framework [43].

**Table 4:** Equity reporting characteristics of included studies

**Equity Reporting Characteristics**	**Count (Percentage)** *N* = 68
Included reviews that define equity/inequity	5 (7.4)
Included reviews with “equit*” or “inequit*” mentioned in the:	
Title	7 (10.3)
Abstract	36 (50.0)
Introduction	32 (44.4)
Methods	20 (27.8)
Results	28 (38.9)
Discussion	39 (54.2)
Conclusion	29 (40.3)
Introduction and/or Discussion/Conclusion Only	19 (26.4)
In All Sections	9 (13.2)
Frequency of “equit*” or “inequit*” mentions in included reviews:	
Maximum	120
Minimum	1
Average	11.9
Mode	2

### Quality assessment

The ROBIS quality appraisal tool was used to assess the included systematic reviews. Majority of the included reviews presented with low to moderate risk of bias. In domain 1 (eligibility criteria), four reviews showed high risk of bias. Two studies showed a high risk of bias for domain 2 (identification and selection) and 12 studies showed a high risk of bias in data collection and appraisal (domain 3). Synthesis and findings (domain 4) and the final overall review of risk of bias only included one article in each with a high risk of bias. The specific scoring of each domain for each included systematic review can be seen in Supplementary file 3.

## Discussion

This umbrella review identified and summarized practices within prenatal healthcare services as they related to equity/inequity, consolidated barriers and facilitators of equity/inequity factors and summarized how these factors influence the prenatal patient experience or health outcomes when accessing/using health services, globally. The included studies represent 20 different countries. In addition to reporting on types and reasons for inequities as described in the included studies, this review consolidates practices that are suggested to facilitate the conditions necessary for health equity in prenatal care (e.g., CHWs, home visitation programs, CCT programs, virtual care options, and cross-cultural training). Additionally, this review explored how equity is presented in each of the systematic reviews, and if the authors of each review provided a working definition or conceptualization of the term.

Our study aligns with recent literature highlighting how inequities lead to suboptimal healthcare for prenatal patients [110–121]. For example, studies investigating access and uptake of prenatal screening services in Canada and New Zealand have identified similar challenges for patients in navigating services. This includes cost of services, remote living, low maternal age, being an ethnic minority, or having a recent immigrant status [110, 121]. In both countries, coverage of basic prenatal screening services is publicly insured for residents [110, 121]. Comparable to our findings, patients lacked knowledge and awareness of available services which was an inherent barrier of accessing care [110, 121]. Disparities in prenatal healthcare have been reported in urban areas in Southern Brazil and rural areas in China, such as the inadequate use and uptake of prenatal supplements (e.g., folic acid or iron) to support the health and development of birthing individuals and their fetus [112, 113, 118]. Indeed, the study in China by Liu et al. reported that despite government recommendations, there was a barrier to uptake of prenatal supplements by pregnant women that had lower levels of education, were an ethnic minority, or were unemployed [112]. A study by Yaya et al. conducted in rural areas of Nigeria uncovered the challenge of gender inequality in accessing healthcare services. Similar to our review, they identified the cultural norm of women having less decision-making power in a relationship and therefore were restricted in accessing quality care by their partner, usually identified as a man [116]. Our review identified that patient-centred care influenced patients’ satisfaction with the care they received. Complementing this finding, a recent study by King et al. found that education and a non-white ethnicity were inversely related to the perceived quality of patient-centred care in a cohort of prenatal patients at a provincial health centre in Canada [122].

This umbrella review also identified facilitators to health equity that led to a greater perceived quality of prenatal healthcare. Although these findings are not as common, recent literature has identified strategies towards achieving health equity in prenatal care [123, 124]. An established prenatal care program in Mexico, a low-middle income country, which targets populations from rural areas with low SES used shared-care between general practitioners, obstetricians, and other specialized health professionals, to ensure a multidisciplinary approach to care, similar to the team-based findings from our review [123]. In the United States of America, a high income country, a study evaluated the effect of trauma-informed care for adolescents receiving prenatal care services at an established adolescent maternity program and found that this strategy led to equitable pregnancy outcomes across racial and ethnic groups, which is comparable to our findings of cross-cultural training for professionals as a strategy to reduce patient anxiety [124].

A gap in our findings was the association between equity/inequity and the implementation climate of practices, which is important to consider for longevity and sustainability of equitable practices [125]. Implementation climate is defined by the surrounding context of where an intervention is to be incorporated; this can include the people, the physical environment, or social or cultural norms [126, 127]. Cultural norms of the implementation climate should be a priori of consideration when establishing how to implement a practice and how inequities may play a role. For example, research from China, Nigeria, and South Africa have investigated the SES of different regions and how this affected the adoption of prenatal healthcare services. The studies depicted that generalizability, spread and scale, are not always possible [111, 112, 128]. Interestingly, Linhares et al. found an inverse inequality distribution where supplements had a greater uptake in urban Southern Brazilian prenatal populations of low income or education level, which depicts how context matters [113]. A recent United States ethnography study of the clinical environment in prenatal care discussed the difference in site specific factors for care that led to differing perspectives of service by patients and healthcare professionals. For example, waiting time was a great disruption in the patient journey. Those who were of low SES, non-white, often of immigrant status or non-English speaking were expected to accommodate their own schedules to the demands of health service centres [115]. A timely example of an implementation climate which influences equitable access to prenatal care is the COVID-19 pandemic. During the pandemic, healthcare services related to obstetrics and gynecology were overlooked, leading to an increase in prenatal morbidity, mortality, and an overall decline in wellbeing [6, 129, 130]. There is limited data in the literature to explain the effects of inequity on access and use of care for this population during and since the COVID-19 pandemic [131]. This umbrella review provides a global perspective of how equity/inequity may influence prenatal care; it is important to consider how the context and implementation climate of different countries plays a role in this influence.

As part of the review, we also examined how equity was reported. Surprisingly, the majority of studies did not define equity and none defined inequity, which adds to the confusion of the use of the terms and how they may be perceived by different researchers and decision-makers [29, 30]. The studies that did define equity [46, 47, 79, 89, 94] were quite consistent; they each mentioned terminology surrounding the inclusion of every person or population including those that are vulnerable or disadvantaged, and the necessity of healthcare to be fair. The inconsistency of definitions became apparent when discussing what constructs were recognized as factors of equity and the spectrum from inequity to equity. Most of the included studies did not use an equity related framework to guide their methods which contributes to the variability in reporting. A relevant study by Hartwell and colleagues from 2022 explored equity reporting characteristics of systematic reviews and meta-analyses that focused on the COVID-19 pandemic, and maternal and childbirth outcomes [131]. This study also used PROGRESS-Plus as a guiding framework and only identified factual relations between outcomes and equity factors. Our umbrella review presents data prior to and during the pandemic, and narrows its focus to the prenatal population to enhance the specificity of how equity influences care in this population.

### Limitations

There are limitations to this umbrella review. We limited our inclusion criteria to English language studies only, which was a decision made due to resource constraints. Prior studies have identified that this limitation does not lead to significant bias within medical research [132]. We also limited our inclusion criteria to studies that mentioned equit*/inequit* because Cochrane’s PRISMA-Equity checklist identifies ‘equity’ in the title as a category [31]. We extended this category to anywhere in the article. Articles that discuss equity without using the term explicitly may have been missed. As we only identified five explicit definitions, this means that there could still be much discrepancy of the use of the term ‘equity’ or ‘inequity’ in healthcare and research, adding to the challenge of effective goal setting and action in health systems change [29, 30]. Further, our analysis of these terms did not separate equity and inequity; the combined analysis may impede the clarity of which of these terms were featured more or less in the studies.

We used a maximum variation technique in our analysis to ensure we captured patterns that emerged within and across the studies, which presented great heterogeneity in context and settings. With this technique, we did not correlate themes specific to context, rather across them. To overcome this barrier, we have provided details on countries which the included studies analyzed to provide insight into context relevant to our data (Supplemental file 4). Additionally, a challenge we faced was extracting data specific to the prenatal period as many of our studies ranged from prenatal specific populations to general unspecified populations and maternal healthcare more broadly. During data extraction, we ensured to only extract data that was relevant to pregnancy before delivery. Data extracted must have identified the population of focus as prenatal. When this was not possible, we did include data that was applicable across the perinatal period, from conception to following birth, which still included a prenatal population. We treated this data in the same way, although Supplementary file 4 does identify which systematic reviews have a prenatal only population.

## Conclusions

In this umbrella review, we explored reported barriers and facilitators to health equity/inequity across the globe and their impact on prenatal care and subsequently infant health and development. The review highlights how equity/inequity influences prenatal patients’ access and use of care within prenatal healthcare practices and collates potential solutions to gaps in health equity for this population. The findings highly overlapped across PROGRESS-Plus equity factors and the barriers and facilitators that we identified are likely much more complex and intertwined [133]. This study adds value to the literature as it shows how current innovations, some of which are common across the globe, are utilized to overcome barriers to achieving equity. The data also speaks to how barriers and potential facilitators or solutions are common across countries. Decision-makers and knowledge-users from across the globe, including healthcare professionals, healthcare administrators, and policy-makers, can apply these findings in their own contexts to improve equity in the access and use of prenatal healthcare services.

### Supplementary Information

**Additional file 1.** Prisma-Equity Checklist. Completed PRISMA-Equity checklist to guide the umbrella review.

**Additional file 2.** Search Strategy. Complete search strategy for all electronic data bases searched for this review.

**Additional file 3.** ROBIS Quality Appraisal of Included Studies. Complete list of included studies in umbrella review with ROBIS quality appraisal scoring.

**Additional file 4.** List of included studies and study characteristics. Complete list of included studies in umbrella review and study characteristics (author, year of publication, title, aim, countries of studies analyzed within included reviews, population, health services focus, outcomes, funding).

**Additional file 5.** List of excluded studies and reason for exclusion. Complete list of excluded studies during search and screening process with reason for exclusion.

## Data Availability

No datasets were generated or analysed during the current study.

## Abbreviations

CCT: Conditional cash transfer
CHW: Community health worker
COVID-19: Coronavirus disease of 2019
LGBTQ2S +: Lesbian, Gay, Bisexual, Transgender, Queer or Questioning, Two-Spirit, Plus
mhealth: Mobile-health
PRISMA-Equity: Preferred Reporting Items for Systematic Reviews and Meta-Analyses—Equity
PROGRESS-Plus: Place of residence, Race/ethnicity/culture/language, Occupation, Gender/sex, Religion, Education, Socioeconomic status, Social capital, Plus
ROBIS: Risk of Bias Assessment Tool for Systematic Reviews
SES: Socioeconomic status

## References

[1] Higginbottom GM, Safipour J, Yohani S, O’Brien B, Mumtaz Z, Paton P (2016). An ethnographic investigation of the maternity healthcare experience of immigrants in rural and urban Alberta, Canada. BMC Pregnancy Childbirth.

[2] Fernandez Turienzo C, Newburn M, Agyepong A, Buabeng R, Dignam A, Abe C (2021). Addressing inequities in maternal health among women living in communities of social disadvantage and ethnic diversity. BMC Public Health.

[3] Sharma S, Kolahdooz F, Launier K, Nader F, June Yi K, Baker P (2016). Canadian Indigenous womens perspectives of maternal health and health care services: a systematic review. Divers Equal Health Care.

[4] Kringos DS, Boerma WG, Bourgueil Y, Cartier T, Hasvold T, Hutchinson A (2010). The European primary care monitor: structure, process and outcome indicators. BMC Fam Pract.

[5] England N, Improvement N. Equity and equality: guidance for local maternity systems. 2021.

[6] Davenport MH, Meyer S, Meah VL, Strynadka MC, Khurana R (2020). Moms are not OK: COVID-19 and maternal mental health. Front Glob Womens Health.

[7] WHO. Health topics: maternal health. 2021. Available from: https://www.who.int/health-topics/maternal-health#tab=tab_1. [cited 2021 Dec 15].

[8] Buultjens M, Farouque A, Karimi L, Whitby L, Milgrom J, Erbas B (2021). The contribution of group prenatal care to maternal psychological health outcomes: a systematic review. Women and Birth..

[9] Yan J (2017). The effects of prenatal care utilization on maternal health and health behaviors. Health Econ.

[10] McDonald S, Kehler H, Bayrampour H, Fraser-Lee N, Tough S (2016). Risk and protective factors in early child development: results from the All Our Babies (AOB) pregnancy cohort. Res Dev Disabil.

[11] Petitclerc A, Richard, Tremblay E. Childhood disruptive behaviour disorders: review of their origin, development, and prevention. Can J Psychiatry. 2009;54(4):222–31. 10.1177/070674370905400403 19321028

[12] Berry OO, Tobón AL, Wanjikũ, Njoroge FM (1920). Social determinants of health: the impact of racism on early childhood mental health. Curr Psychiatry Rep.

[13] Enns JE, Nickel NC, Chartier M, Chateau D, Campbell R, Phillips-Beck W (2021). An unconditional prenatal income supplement is associated with improved birth and early childhood outcomes among First Nations children in Manitoba, Canada: a population-based cohort study. BMC Pregnancy Childbirth.

[14] Puthussery S (2016). Perinatal outcomes among migrant mothers in the United Kingdom: is it a matter of biology, behaviour, policy, social determinants or access to health care?. Best Pract Res Clin Obstet Gynaecol.

[15] Evans A, Ray JG, Austin PC, Lu H, Gandhi S, Guttmann A (2023). Receipt of adequate prenatal care for privately sponsored versus government-assisted refugees in Ontario, Canada: a population-based cohort study. CMAJ.

[16] Varner CE, Park AL, Ray JG (2023). Maternal emergency department use before pregnancy and infant emergency department use after birth. JAMA Netw Open.

[17] Dragonas T, Christodoulou GN (1998). Prenatal Care. Clin Psychol Rev..

[18] Crear-Perry J, Correa-De-Araujo R, Lewis Johnson T, Mclemore MR, Neilson E, Wallace M (2021). Social and structural determinants of health inequities in maternal health. J Womens Health.

[19] Langell JT (2021). Evidence-based medicine: a data-driven approach to lean healthcare operations. Int J Healthc Manag.

[20] Michaels JA (2021). Potential for epistemic injustice in evidence-based healthcare policy and guidance. J Med Ethics.

[21] Buxton M, Hanney S (1996). How can payback from health services research be assessed? The strategic importance of assessing payback. J Health Serv Res.

[22] Public Health Agency of Canada. Government of Canada. 2019. Family-centred maternity and newborn care: National guidelines. Available from: https://www.canada.ca/en/public-health/services/publications/healthy-living/maternity-newborn-care-guidelines-chapter-3.html. [cited 2022 Jan 27].

[23] Shmerling A, Hoss M, Malam N, Staton EW, Lyon C (2022). Prenatal care via telehealth. Prim Care Clin Office Pract.

[24] Booth A. EVIDENT guidance for reviewing the evidence: a compendium of methodological literature and websites. University of Sheffield. 2016. Available from: www.academia.edu/21598179/EVIDENT_Guidance_for_Reviewing_the_Evidence_a_compendium_of_methodological_literature_and_websites. [cited 2024 Mar 6].

[25] Tsagris M, Fragkos KC. Umbrella reviews, overviews of reviews, and meta-epidemiologic studies: similarities and differences. In: Umbrella reviews: evidence synthesis with overviews of reviews and meta-epidemiologic studies. Switzerland: Springer International Publishing; 2016. p. 43–54.

[26] Aromataris E, Fernandez RS, Godfrey C, Holly C, Khalil H. Methodology for JBI umbrella reviews. 2014.

[27] Grant MJ, Booth A (2009). A typology of reviews: an analysis of 14 review types and associated methodologies. Health Inform Lib J.

[28] Aromataris E, Fernandez R, Godfrey CM, Holly C, Khalil H, Tungpunkom P (2015). Summarizing systematic reviews: methodological development, conduct and reporting of an umbrella review approach. Int J Evid Based Healthc.

[29] Braveman P, Gruskin S (2003). Defining equity in health. J Epidemiol Community Health.

[30] Braveman P, Arkin E, Orleans T, Proctor D, Acker J, Plough A (2018). What is health equity?. Behav Sci Policy.

[31] Welch V, Petticrew M, Tugwell P, Moher D, O’Neill J, Waters E (2012). PRISMA-equity 2012 extension: reporting guidelines for systematic reviews with a focus on health equity. PLoS Med.

[32] O’Neill J, Tabish H, Welch V, Petticrew M, Pottie K, Clarke M (2014). Applying an equity lens to interventions: Using PROGRESS ensures consideration of socially stratifying factors to illuminate inequities in health. J Clin Epidemiol.

[33] Ladak Z, Grewal N, Small S, Hemani M, Leber A, Kim O, et al. PROSPERO International prospective register of systematic reviews Citation. 2022. Available from: https://www.crd.york.ac.uk/prospero/display_record.php?ID=CRD42022301574.

[34] Montori VM, Wilczynski NL, Morgan D, Haynes RB (2005). Optimal search strategies for retrieving systematic reviews from Medline: analytical survey. Br Med J.

[35] Wilczynski NL, Haynes RB (2007). EMBASE search strategies achieved high sensitivity and specificity for retrieving methodologically sound systematic reviews. J Clin Epidemiol.

[36] Veritas Health Innovation, Melbourne, Australia. Covidence systematic review software. Available from: www.covidence.org. [cited 2023 Nov 16].

[37] Patton MQ. Designing Qualitative Studies. In: Qualitative research & evaluation methods: Integrating theory and practice. Sage publications. 2014;(4):244–326

[38] Hsieh HF, Shannon SE (2005). Three approaches to qualitative content analysis. Qual Health Res.

[39] Zhang Y, Wildemuth BM (2005). Qualitative analysis of content. Hum Brain Mapp.

[40] Whiting P, Savović J, Higgins JPT, Caldwell DM, Reeves BC, Shea B (2016). ROBIS: a new tool to assess risk of bias in systematic reviews was developed. J Clin Epidemiol.

[41] Moncrieff G, Finlayson K, Cordey S, McCrimmon R, Harris C, Barreix M (2021). First and second trimester ultrasound in pregnancy: a systematic review and metasynthesis of the views and experiences of pregnant women, partners, and health workers. PLoS One.

[42] Kirubarajan A, Barker LC, Leung S, Ross LE, Zaheer J, Park B (2022). LGBTQ2S+ childbearing individuals and perinatal mental health: a systematic review. BJOG.

[43] Lyall V, Wolfson L, Reid N, Poole N, Moritz KM, Egert S (2021). “The problem is that we hear a bit of everything … ”: a qualitative systematic review of factors associated with alcohol use, reduction, and abstinence in pregnancy. Int J Environ Res Public Health.

[44] Toh RKC, Shorey S (2023). Experiences and needs of women from ethnic minorities in maternity healthcare: a qualitative systematic review and meta-aggregation. Women Birth.

[45] Murray SF, Hunter Msc BM, Bisht R, Ensor T, Bick D (2012). Demand-side financing measures to increase maternal health service utilisation and improve health outcomes: a systematic review of evidence from low-and middle-income countries Executive summary Background. JBI Libr Syst Rev.

[46] McCollum R, Gomez W, Theobald S, Taegtmeyer M (2016). How equitable are community health worker programmes and which programme features influence equity of community health worker services? A systematic review. BMC Public Health..

[47] Blanchard AK, Prost A, Houweling TAJ (2019). Effects of community health worker interventions on socioeconomic inequities in maternal and newborn health in low-income and middle-income countries: a mixed-methods systematic review. BMJ Glob Health.

[48] Banke-Thomas A, Wright K, Collins L. Assessing geographical distribution and accessibility of emergency obstetric care in sub- Saharan Africa: a systematic review. J Glob Health. 2019;9(1):010414. 10.7189/jogh.09.010414 PMC6304172 30603080

[49] Watson H, Harrop D, Walton E, Young A, Soltani H (2019). A systematic review of ethnic minority women’s experiences of perinatal mental health conditions and services in Europe. PLoS One.

[50] Dahab R, Sakellariou D (2020). Barriers to accessing maternal care in low income countries in Africa: a systematic review. Int J Environ Res Public Health.

[51] Chando S, Tong A, Howell M, Dickson M, Craig JC, DeLacy J (2021). Stakeholder perspectives on the implementation and impact of Indigenous health interventions: a systematic review of qualitative studies. Health Expect.

[52] Sidze EM, Wekesah FM, Kisia L, Abajobir A (2022). Inequalities in access and utilization of maternal, newborn and child health services in sub-Saharan Africa: a special focus on urban settings. Matern Child Health J.

[53] Say L, Raine R (2007). A systematic review of inequalities in the use of maternal health care in developing countries: examining the scale of the problem and the importance of context. Bull World Health Org.

[54] Lee ACC, Lawn JE, Cousens S, Kumar V, Osrin D, Bhutta ZA (2009). Linking families and facilities for care at birth: what works to avert intrapartum-related deaths?. Int J Gynecol Obstet.

[55] Lewin S, Munabi-Babigumira S, Glenton C, Daniels K, Bosch-Capblanch X, van Wyk BE, et al. Lay health workers in primary and community health care for maternal and child health and the management of infectious diseases. Cochrane Database Syst Rev. 2010;2010(3):CD004015. 10.1002/14651858.CD004015.pub3 PMC6485809 20238326

[56] Byrne A, Hodge A, Jimenez-Soto E, Morgan A (2014). What works? Strategies to increase reproductive, maternal and child health in difficult to access mountainous locations: a systematic literature review. PLoS One.

[57] Gopalan SS, Mutasa R, Friedman J, Das A (2014). Health sector demand-side financial incentives in low- and middle-income countries: a systematic review on demand- and supply-side effects. Soc Sci Med.

[58] Hunter BM, Murray SF (2017). Demand-side financing for maternal and newborn health: What do we know about factors that affect implementation of cash transfers and voucher programmes?. BMC Pregnancy Childbirth.

[59] Wekesah FM, Mbada CE, Muula AS, Kabiru CW, Muthuri SK, Izugbara CO (2016). Effective non-drug interventions for improving outcomes and quality of maternal health care in sub-Saharan Africa: a systematic review. Syst Rev.

[60] Palmer MJ, Henschke N, Bergman H, Villanueva G, Maayan N, Tamrat T, et al. Targeted client communication via mobile devices for improving maternal, neonatal, and child health. Cochrane Database Syst Rev. 2020;2020(8):CD013679. 10.1002/14651858.CD013679 PMC8477611 32813276

[61] Hollowell J, Oakley L, Kurinczuk JJ, Brocklehurst P, Gray R (2011). The effectiveness of antenatal care programmes to reduce infant mortality and preterm birth in socially disadvantaged and vulnerable women in high-income countries: a systematic review. BMC Pregnancy Childbirth.

[62] Mcarthur A, Lockwood C (2013). Maternal mortality in Cambodia, Thailand, Malaysia and Sri Lanka: a systematic review of local and national policy and practice initiatives Executive summary Background. JBI Database Syst Rev Implement Rep.

[63] Lassi ZS, Middleton PF, Bhutta ZA, Crowther C (2016). Strategies for improving health care seeking for maternal and newborn illnesses in low- and middle-income countries: a systematic review and meta-analysis. Global Health Act.

[64] Walker RC, Graham A, Palmer SC, Jagroop A, Tipene-Leach DC (2019). Understanding the experiences, perspectives and values of indigenous women around smoking cessation in pregnancy: systematic review and thematic synthesis of qualitative studies. Int J Equity Health.

[65] Rowe RE, Garcia J (2003). Social class, ethnicity and attendance for antenatal care in the United Kingdom: a systematic review. J Public Health Med.

[66] Rowe RE, Garcia J, Davidson LL (2004). Social and ethnic inequalities in the offer and uptake of prenatal screening and diagnosis in the UK: a systematic review. Public Health.

[67] Feijen-De Jong EI, Jansen DE, Baarveld F, Van Der Schans CP, Schellevis FG, Reijneveld SA (2012). Determinants of late and/or inadequate use of prenatal healthcare in high-income countries: a systematic review. Eur J Public Health.

[68] Yu J (2012). A systematic review of issues around antenatal screening and prenatal diagnostic testing for genetic disorders: women of Asian origin in western countries. Health Soc Care Commun.

[69] Mutambudzi M, Meyer JD, Reisine S, Warren N (2017). A review of recent literature on materialist and psychosocial models for racial and ethnic disparities in birth outcomes in the US, 2000–2014. Ethn Health.

[70] Prady SL, Endacott C, Dickerson J, Bywater TJ, Blower SL (2021). Inequalities in the identification and management of common mental disorders in the perinatal period: an equity focused reanalysis of a systematic review. Public Lib Sci.

[71] Balaam MC, Akerjordet K, Lyberg A, Kaiser B, Schoening E, Fredriksen AM (2013). A qualitative review of migrant women’s perceptions of their needs and experiences related to pregnancy and childbirth. J Adv Nurs.

[72] Knight HE, Self A, Kennedy SH (2013). Why are women dying when they reach hospital on time? A systematic review of the “Third Delay”. PLoS One.

[73] Small R, Roth C, Raval M, Shafiei M, Korfker D, Heaman M (2014). Immigrant and non-immigrant women’s experiences of maternity care: a systematic and comparative review of studies in five countries. BMC Pregnancy Childbirth.

[74] Higginbottom GMA, Evans C, Morgan M, Bharj KK, Eldridge J, Hussain B (2019). Experience of and access to maternity care in the UK by immigrant women: a narrative synthesis systematic review. BMJ Open.

[75] Fair F, Raben L, Watson H, Vivilaki V, van den Muijsenbergh M, Soltani H (2020). Migrant women’s experiences of pregnancy, childbirth and maternity care in European countries: a systematic review. PLoS One.

[76] Firdous T, Darwin Z, Hassan SM (2020). Muslim women’s experiences of maternity services in the UK: qualitative systematic review and thematic synthesis. BMC Pregnancy Childbirth.

[77] Kyei-Nimakoh M, Carolan-Olah M, McCann TV (2017). Access barriers to obstetric care at health facilities in sub-Saharan Africa-a systematic review. Syst Rev.

[78] Ames HMR, Glenton C, Lewin S, Tamrat T, Akama E, Leon N (2019). Clients’ perceptions and experiences of targeted digital communication accessible via mobile devices for reproductive, maternal, newborn, child, and adolescent health: a qualitative evidence synthesis. Cochrane Database Syst Rev.

[79] Målqvist M, Hoa DTP, Thomsen S (2012). Causes and determinants of inequity in maternal and child health in Vietnam. BMC Public Health.

[80] De Maio FG (2010). Immigration as pathogenic: a systematic review of the health of immigrants to Canada. Int J Equity Health.

[81] Ag Ahmed MA, Gagnon MP, Hamelin-Brabant L, Mbemba GIC, Alami H (2017). A mixed methods systematic review of success factors of mhealth and telehealth for maternal health in Sub-Saharan Africa. Mhealth.

[82] Byrne A, Hodge A, Jimenez-Soto E, Morgan A (2013). Looking beyond supply: A systematic literature review of demand-side barriers to health service utilization in the mountains of Nepal. Asia Pac J Public Health.

[83] Muralidharan A, Fehringer J, Pappa S, Rottach E, Das M, Mandal M (2015). Transforming gender norms, roles, and power dynamics for better health: evidence from a systematic review of gender-integrated health programs in low- and middle-income countries.

[84] Benova L, Campbell OMR, Ploubidis GB (2014). Socio-economic gradients in maternal and child health-seeking behaviours in Egypt: systematic literature review and evidence synthesis. PLoS One.

[85] Mengist B, Desta M, Tura AK, Habtewold TD, Abajobir A (2021). Maternal near miss in Ethiopia: protective role of antenatal care and disparity in socioeconomic inequities: a systematic review and meta-analysis. Int J Afr Nurs Sci.

[86] Darmstadt GL, Lee ACC, Cousens S, Sibley L, Bhutta ZA, Donnay F (2009). 60 Million non-facility births: who can deliver in community settings to reduce intrapartum-related deaths?. Int J Gynecol Obstet.

[87] Ehiri JE, Gunn JKL, Center KE, Li Y, Rouhani M, Ezeanolue EE (2014). Training and deployment of lay refugee/internally displaced persons to provide basic health services in camps: a systematic review. Global Health Act.

[88] Bovill M, Chamberlain C, Bar-Zeev Y, Gruppetta M, Gould GS (2019). Ngu - Ng - gi - La - nha (to exchange) knowledge. How is Aboriginal and Torres Strait Islander people’s empowerment being upheld and reported in smoking cessation interventions during pregnancy: a systematic review. Austr J Prim Health..

[89] Beatson R, Molloy C, Perini N, Harrop C, Goldfeld S (2021). Systematic review: an exploration of core componentry characterizing effective sustained nurse home visiting programs. J Adv Nurs.

[90] Magwood O, Kpadé V, Thavorn K, Oliver S, Mayhew AD, Pottie K (2019). Effectiveness of home-based records on maternal, newborn and child health outcomes: a systematic review and meta-analysis. PLoS One.

[91] Magwood O, Kpadé V, Afza R, Oraka C, McWhirter J, Oliver S (2019). Understanding women’s, caregivers’, and providers’ experiences with home-based records: a systematic review of qualitative studies. PLoS One.

[92] Çalışkan Z, Kılıç D, Öztürk S, Atılgan E (2015). Equity in maternal health care service utilization: a systematic review for developing countries. Int J Public Health.

[93] Tokhi M, Comrie-Thomson L, Davis J, Portela A, Chersich M, Luchters S (2018). Involving men to improve maternal and newborn health: a systematic review of the effectiveness of interventions. PLoS One.

[94] Victoria C, Barros F, Assunção M, Restrepo-Méndez M, Matijasevich A, Martorell R (2012). Scaling up maternal nutrition programs to improve birth outcomes: a review of implementation issues. Food Nutr Bull.

[95] Cormick G, Betran A, Romero I, Lombardo C, Gulmezoglu A, Ciapponi A (2019). Global inequities in dietary calcium intake duringpregnancy: a systematic review and meta-analysis. BJOG.

[96] Ogundele OJ, Pavlova M, Groot W (2020). Socioeconomic inequalities in reproductive health care services across Sub-Saharan Africa. A systematic review and meta-analysis. Sex Reprod Healthc.

[97] Vanstone M, Cernat A, Majid U, Trivedi F, De Freitas C (2019). Perspectives of pregnant people and clinicians on noninvasive prenatal testing: a systematic review and qualitative meta-synthesis. Ont Health Technol Assess Ser.

[98] Jhaveri R, Yee LM, Antala S, Murphy M, Grobman WA, Shah SK (2021). Responsible inclusion of pregnant individuals in eradicating HCV. Hepatology.

[99] Lagarde M, Haines A, Palmer N (2007). Conditional cash transfers for improving uptake of health interventions in low-and middle-income countries: a systematic review. JAMA.

[100] Lagarde M, Palmer N (2008). The impact of user fees on health service utilization in low- and middle-income countries: how strong is the evidence?. Bull World Health Organ.

[101] Glassman A, Duran D, Fleisher L, Singer D, Sturke R, Angeles G (2013). Impact of conditional cash transfers on maternal and newborn health. J Health Popul Nutr.

[102] Målqvist M, Yuan B, Trygg N, Selling K, Thomsen S (2013). Targeted interventions for improved equity in maternal and child health in low- and middle-income settings: a systematic review and meta-analysis. PLoS One.

[103] Yuan B, Målqvist M, Trygg N, Qian X, Ng N, Thomsen S (2014). What interventions are effective on reducing inequalities in maternal and child health in low- and middle-income settings? A systematic review. BMC Public Health.

[104] Hunter BM, Harrison S, Portela A, Bick D (2017). The effects of cash transfers and vouchers on the use and quality of maternity care services: a systematic review. PLoS One.

[105] Yaya S, Sanogo AN (2019). Universal health coverage and facilitation of equitable access to care in Africa: a systematic review. Front Public Health.

[106] Widmer M, Betran AP, Merialdi M, Requejo J, Karpf T (2011). The role of faith-based organizations in maternal and newborn health care in Africa. Int J Gynecol Obstet.

[107] Lassi ZS, Haider BA, Bhutta ZA (2012). Community-based intervention packages for reducing maternal morbidity and mortality and improving neonatal outcomes. J Dev Effect.

[108] Chamberlain C, Mara-Eves A, Oliver S, Caird J, Perlen S, Eades S (2013). Psychosocial interventions for supporting women to stop smoking in pregnancy. Cochrane Database Syst Rev.

[109] Saad A, Magwood O, Aubry T, Alkhateeb Q, Hashmi SS, Hakim J (2021). Mobile interventions targeting common mental disorders among pregnant and postpartum women: an equity-focused systematic review. PLoS One.

[110] Payne O, Pillai A, Wise M, Stone P (2017). Inequity in timing of prenatal screening in New Zealand: who are our most vulnerable?. Aust N Z J Obstet Gynaecol.

[111] Ngandu NK, Van Malderen C, Goga A, Speybroeck N (2017). Wealth-related inequality in early uptake of HIV testing among pregnant women: an analysis of data from a national cross-sectional survey, South Africa. BMJ Open.

[112] Liu M, Chen J, Liu J, Zhang S, Wang Q, Shen H (2017). Socioeconomic inequality in periconceptional folic acid supplementation in China: a census of 0.9 million women in their first trimester of pregnancy. BMC Pregnancy Childbirth.

[113] Linhares AO, da Linhares RS, Cesar JA (2017). Inequality in iron sulfate supplementation among pregnant women in Southern Brazil. Rev Bras Epidemiol.

[114] Noah AJ, Yang TC, Wang WL (2018). The black-white disparity in sexually transmitted diseases during pregnancy: how do racial segregation and income inequality matter?. Sex Transm Dis.

[115] Andaya E (2019). Race-ing time: clinical temporalities and inequality in public prenatal care. Med Anthropol.

[116] Yaya S, Okonofua F, Ntoimo L, Udenige O, Bishwajit G (2019). Gender inequity as a barrier to women’s access to skilled pregnancy care in rural Nigeria: a qualitative study. Int Health.

[117] Vedam S, Stoll K, Taiwo TK, Rubashkin N, Cheyney M, Strauss N (2019). The Giving Voice to Mothers study: inequity and mistreatment during pregnancy and childbirth in the United States. Reprod Health.

[118] Miranda VIA, da Silva Dal Pizzol T, Silveira MPT, Mengue SS, da Silveira MF, Lutz BH (2019). The use of folic acid, iron salts and other vitamins by pregnant women in the 2015 Pelotas birth cohort: is there socioeconomic inequality?. BMC Public Health.

[119] Chirwa GC, Mazalale J, Likupe G, Nkhoma D, Chiwaula L, Chintsanya J (2019). An evolution of socioeconomic related inequality in teenage pregnancy and childbearing in Malawi. PLoS One.

[120] Vilda D, Wallace M, Dyer L, Harville E, Theall K (2019). Income inequality and racial disparities in pregnancy-related mortality in the US. SSM Popul Health.

[121] Hayeems RZ, Campitelli M, Ma X, Huang T, Walker M, Guttmann A (2015). Rates of prenatal screening across health care regions in Ontario, Canada: a retrospective cohort study. CMAJ Open.

[122] King A, Piccinini-Vallis H (2023). Patient-perceived patient-centeredness during pregnancy. J Obstet Gynaecol Can.

[123] de la Bermudez Rojas ML, Medina Jimenez V, Manzanares Cuadros JI, Diaz Martínez DA, Padilla Raygoza N, Lara Lona E (2023). Universal prenatal screening: a initiative from Guanajuato, Mexico to improve equity in perinatal healthcare. Front Med (Lausanne).

[124] Noroña-Zhou AN, Ashby BD, Richardson G, Ehmer A, Scott SM, Dardar S (2023). Rates of preterm birth and low birth weight in an adolescent obstetric clinic: achieving health equity through trauma-informed care. Health Equity.

[125] Hamza DM, Regehr G (2021). Eco-normalization: evaluating the longevity of an innovation in context. Acad Med.

[126] Damschroder LJ, Reardon CM, OpraWiderquist MA, Lowery J (2022). Conceptualizing outcomes for use with the Consolidated Framework for Implementation Research (CFIR): the CFIR Outcomes Addendum. Implement Sci.

[127] Damschroder LJ, Aron DC, Keith RE, Kirsh SR, Alexander JA, Lowery JC (2009). Fostering implementation of health services research findings into practice: a consolidated framework for advancing implementation science. Implement Sci.

[128] Muhammad FM, Majdzadeh R, Nedjat S, Sajadi HS, Parsaeian M (2020). Socioeconomic inequality in intermittent preventive treatment using Sulphadoxine pyrimethamine among pregnant women in Nigeria. BMC Public Health.

[129] Barbosa-Leiker C, Smith CL, Crespi EJ, Brooks O, Burduli E, Ranjo S (2021). Stressors, coping, and resources needed during the COVID-19 pandemic in a sample of perinatal women. BMC Pregnancy Childbirth.

[130] Sarwer A, Javed B, Soto EB, ur Mashwani ZR (2020). Impact of the COVID-19 pandemic on maternal health services in Pakistan. Int J Health Plan Manag.

[131] Hartwell M, Lin V, Gatewood A, Sajjadi NB, Garrett M, Reddy AK (2022). Health disparities, COVID-19, and maternal and childbirth outcomes: a meta-epidemiological study of equity reporting in systematic reviews. J Matern Fetal Neonatal Med.

[132] Morrison A, Polisena J, Husereau D, Moulton K, Clark M, Fiander M (2012). The effect of English-language restriction on systematic review-based meta-analyses: a systematic review of empirical studies. Int J Technol Assess Health Care.

[133] Harris LM, Forson-Dare Z, Gallagher PG (2021). Critical disparities in perinatal health—understanding risks and changing the outcomes. J Perinatol.

