# Supplementary material for: Equity in prenatal healthcare services globally: an umbrella review
Source: BMC Pregnancy Childbirth. 2024 Mar 11;24:191. doi: 10.1186/s12884-024-06388-0 (PMC10926563; doi:10.1186/s12884-024-06388-0)
Supplement: Supplementary file 1 — Additional file 1. Prisma-Equity Checklist. Completed PRISMA-Equity checklist to guide the umbrella review. [file 12884_2024_6388_MOESM1_ESM.pdf]

PRISMA-E 2012 Checklist

Checklist of Items for Reporting Equity-Focused Systematic Reviews				
**Section**	**Item**	**Standard PRISMA Item**	**Extension for Equity-Focused Reviews**	~~Pg #~~ Section
**Title**				
**Title**	1	Identify the report as a systematic review, meta-analysis, or both. ✓	Identify equity as a focus of the review, if relevant, using the term equity	Title
**Abstract**				
**Structured summary**	2	2. Provide a structured summary including, as applicable: background; objectives; data sources; study eligibility criteria, participants, and interventions; study appraisal and synthesis methods; results; limitations; conclusions and implications of key findings; systematic review registration number. ✓	State research question(s) related to health equity.	Abstract-Background
	2A		Present results of health equity analyses (e.g. subgroup analyses or meta-regression).	Abstract-Results
	2B		Describe extent and limits of applicability to disadvantaged populations of interest.	Abstract-Background, Results, Conclusion
**Introduction**				
**Rationale**	3	Describe the rationale for the review in the context of what is already known. ✓	Describe assumptions about mechanism(s) by which the intervention is assumed to have an impact on health equity.	Intro-Par1+2
	3A		Provide the logic model/analytical framework, if done, to show the pathways through which the intervention is assumed to affect health equity and how it was developed.	N/A
**Objectives**	4	Provide an explicit statement of questions being addressed with reference to participants, interventions, comparisons, outcomes, and study design (PICOS). ✓	Describe how disadvantage was defined if used as criterion in the review (e.g. for selecting studies, conducting analyses or judging applicability).	Intro-Par2+4
	4A		State the research questions being addressed with reference to health equity	Intro-Par4
**Methods**				
**Protocol and registration**	5	Indicate if a review protocol exists, if and where it can be accessed (e.g., Web address), and, if available, provide registration information including registration number. ✓		
**Eligibility criteria**	6	6. Specify study characteristics (e.g., PICOS, length of follow-up) and report characteristics (e.g., years considered, language, publication status) used as criteria for eligibility, giving rationale. ✓	Describe the rationale for including particular study designs related to equity research questions.	Intro-Par3 Methods-Par2, Table1
	6A		Describe the rationale for including the outcomes - e.g. how these are relevant to reducing inequity.	Methods-Par2
**Information sources**	7	Describe all information sources (e.g., databases with dates of coverage, contact with study authors to identify additional studies) in the search and date last searched. ✓	Describe information sources (e.g. health, non-health, and grey literature sources) that were searched that are of specific relevance to address the equity questions of the review.	Methods-Par3
**Search**	8	Present full electronic search strategy for at least one database, including any limits used, such that it could be repeated. ✓	Describe the broad search strategy and terms used to address equity questions of the review.	Methods-Par3
**Study selection**	9	State the process for selecting studies (i.e., screening, eligibility, included in systematic review, and, if applicable, included in the meta-analysis). ✓		
**Data collection process**	10	Describe method of data extraction from reports (e.g., piloted forms, independently, in duplicate) and any processes for obtaining and confirming data from investigators. ✓		
**Data items**	11	List and define all variables for which data were sought (e.g., PICOS, funding sources) and any assumptions and simplifications made. ✓	List and define data items related to equity,where such data were sought (e.g. using PROGRESS-Plus or other criteria, context).	Methods-Par5-8
**Risk of bias in individual studies**	12	Describe methods used for assessing risk of bias of individual studies (including specification of whether this was done at the study or outcome level), and how this information is to be used in any data synthesis. ✓		
**Summary measures**	13	State the principal summary measures (e.g., risk ratio, difference in means).		N/A
**Synthesis of results**	14	Describe the methods of handling data and combining results of studies, if done, including measures of consistency (e.g., I^2) for each meta-analysis. ✓	Describe methods of synthesizing findings on health inequities (e.g. presenting both relative and absolute differences between groups).	Methods-Par5-8
**Risk of bias**	15	15. Specify any assessment of risk of bias that may affect the cumulative evidence		N/A

**across studies**		(e.g., publication bias, selective reporting within studies).		
**Additional analyses**	16	Describe methods of additional analyses (e.g., sensitivity or subgroup analyses, meta-regression), if done, indicating which were pre-specified.	Describe methods of additional synthesis approaches related to equity questions, if done, indicating which were pre-specified	N/A
**Results**				
**Study selection**	17	Give numbers of studies screened, assessed for eligibility, and included in the review, with reasons for exclusions at each stage, ideally with a flow diagram. ✓		
**Study characteristics**	18	For each study, present characteristics for which data were extracted (e.g., study size, PICOS, follow-up period) and provide the citations. ✓	Present the population characteristics that relate to the equity questions across the relevant PROGRESS-Plus or other factors of interest.	Results-Par3 Supp File 4
**Risk of bias within studies**	19	Present data on risk of bias of each study and, if available, any outcome level assessment (see item 12). ✓		
**Results of individual studies**	20	For all outcomes considered (benefits or harms), present, for each study: (a) simple summary data for each intervention group (b) effect estimates and confidence intervals, ideally with a forest plot. ✓		
**Synthesis of results**	21	Present results of each meta-analysis done, including confidence intervals and measures of consistency. ✓	Present the results of synthesizing findings on inequities (see 14).	Results-Par4-14, Tables 2-4
**Risk of bias across studies**	22	Present results of any assessment of risk of bias across studies (see Item 15).		N/A
**Additional analysis**	23	Give results of additional analyses, if done (e.g., sensitivity or subgroup analyses, meta-regression [see Item 16]).	Give the results of additional synthesis approaches related to equity objectives, if done, (see 16).	N/A
**Discussion**				
**Summary of evidence**	24	Summarize the main findings including the strength of evidence for each main outcome; consider their relevance to key groups (e.g., healthcare providers, users, and policy makers). ✓		
**Limitations**	25	Discuss limitations at study and outcome level (e.g., risk of bias), and at review-level (e.g., incomplete retrieval of identified research, reporting bias). ✓		
**Conclusions**	26	Provide a general interpretation of the results in the context of other evidence, and implications for future research. ✓	Present extent and limits of applicability to disadvantaged populations of interest and describe the evidence and logic underlying those judgments.	Conclusion
	26A		Provide implications for research, practice or policy related to equity where relevant (e.g. types of research needed to address unanswered questions).	Conclusion
**Funding**				
**Funding**	27	Describe sources of funding for the systematic review and other support (e.g., supply of data); role of funders for the systematic review. ✓		

From: Source: Welch V, Petticrew M, Tugwell P, Moher D, O'Neill J, Waters E, White H, and the PRISMA-Equity Bellagio Group. (2012) PRISMA-Equity 2012 Extension: Reporting Guidelines for Systematic Reviews with a Focus on Health Equity. PLoS Med 9(10): e1001333. doi:10.1371/journal.pmed.1001333

For more information: http://equity.cochrane.org/equity-extension-prisma
