# Supplementary material for: Equity in prenatal healthcare services globally: an umbrella review
Source: BMC Pregnancy Childbirth. 2024 Mar 11;24:191. doi: 10.1186/s12884-024-06388-0 (PMC10926563; doi:10.1186/s12884-024-06388-0)
Supplement: Supplementary file 2 — Additional file 2. Search Strategy. Complete search strategy for all electronic data bases searched for this review. [file 12884_2024_6388_MOESM2_ESM.pdf]

Search Strategy

CINAHL Plus (EBSCO):
TX (prenatal* OR pre natal* OR antenatal* OR ante natal* OR prepartum OR pre partum OR perinatal* OR peri natal* OR peripartum OR peri partum OR pregnan* OR gestation*)
AND
TX (equit* OR inequit*)
AND
TX (systematic review* OR meta-analys*) OR PT (systematic review* OR meta-analys*)

Medline (OVID):
(prenatal* OR pre natal* OR antenatal* OR ante natal* OR prepartum OR pre partum OR perinatal* OR peri natal* OR peripartum OR peri partum OR pregnan* OR gestation*).mp. and (equit* or inequit*).mp. and ((systematic review* or meta-analys*).pt. or (systematic review* or meta-analys*).mp.)

EMBASE (OVID):
(prenatal* OR pre natal* OR antenatal* OR ante natal* OR prepartum OR pre partum OR perinatal* OR peri natal* OR peripartum OR peri partum OR pregnan* OR gestation*).mp. and (equit* or inequit*).mp. and ((systematic review* or meta-analys*).pt. or (systematic review* or meta-analys*).mp.)

APA PsycInfo (OVID):
(prenatal* OR pre natal* OR antenatal* OR ante natal* OR prepartum OR pre partum OR perinatal* OR peri natal* OR peripartum OR peri partum OR pregnan* OR gestation*).mp. and (equit* or inequit*).mp. and ((systematic review* or meta-analys*).pt. or (systematic review* or meta-analys*).mp.)

IBSS (ProQuest):
(prenatal* OR pre-natal* OR antenatal* OR ante-natal* OR prepartum OR pre-partum OR perinatal* OR peri-natal* OR peripartum OR peri-partum OR pregnan* OR gestation*)
AND
(equit* OR inequit*)
AND
(systematic review* OR meta-analys*)

Cochrane:
(prenatal* OR pre natal* OR antenatal* OR ante natal* OR prepartum OR pre partum OR perinatal* OR peri natal* OR peripartum OR peri partum OR pregnan* OR gestation*):ti,ab,kw and (equit* or inequit*):ti,ab,kw and (systematic review* or meta-analys*):ti,ab,kw
