# Supplementary material for: Equity in prenatal healthcare services globally: an umbrella review
Source: BMC Pregnancy Childbirth. 2024 Mar 11;24:191. doi: 10.1186/s12884-024-06388-0 (PMC10926563; doi:10.1186/s12884-024-06388-0)
Supplement: Supplementary file 3 — Additional file 3. ROBIS Quality Appraisal of Included Studies. Complete list of included studies in umbrella review with ROBIS quality appraisal scoring. [file 12884_2024_6388_MOESM3_ESM.pdf]

ROBIS Quality Appraisal of Included Studies

		**D1: Eligibility Criteria**	**D2: Identification & Selection**	**D3: Data Collection & Appraisal**	**D4: Synthesis & Findings**	**Risk of Bias in Overall Review**
**First Author Last Name**	**Publication Year**	Risk of Bias:<br>Low = 4 and 5<br>Moderate: = 3<br>High: 0 to 2<br><br>*Total Score: 5*	Risk of Bias:<br>Low = 4 and 5<br>Moderate: = 3<br>High: 0 to 2<br><br>*Total Score: 5*	Risk of Bias:<br>Low = 4 and 5<br>Moderate: = 3<br>High: 0 to 2<br><br>*Total Score: 5*	Risk of Bias:<br>Low = 5 and 6<br>Moderate: = 3 and 4<br>High: 0 to 2<br><br>*Total Score: 6*	Risk of Bias:<br>Low = 3<br>Moderate: = 2<br>High: 0 to 1<br><br>*Total Score: 3*
Ahmed	2017	**5**	**5**	**5**	**4**	**2**
Ames	2019	**5**	**5**	**5**	**5**	**2**
Balaam	2013	**3**	**4**	**4**	**3**	**2**
Banke-Thomas	2020	**5**	**5**	**2**	**4**	**3**
Beatson	2014	**5**	**5**	**3**	**6**	**3**
Benova	2021	**3**	**3**	**3**	**5**	**2**
Blanchard	2019	**5**	**4**	**3**	**5**	**2**
Bovill	2019	**5**	**5**	**4**	**6**	**2**
Byrne	2013	**4**	**5**	**3**	**6**	**2**
Byrne	2014	**5**	**4**	**5**	**6**	**2**
Caliskan	2015	**2**	**5**	**3**	**4**	**2**
Chamberlain	2013	**5**	**5**	**5**	**6**	**3**
Chando	2021	**4**	**5**	**3**	**5**	**2**
Cormick	2018	**5**	**5**	**5**	**5**	**2**
Dahab	2020	**5**	**3**	**4**	**6**	**3**
Darmstadt	2009	**5**	**4**	**3**	**4**	**2**
DeMaio	2010	**4**	**4**	**2**	**4**	**2**
Ehiri	2014	**4**	**5**	**5**	**5**	**3**
Fair	2020	**4**	**5**	**4**	**3**	**2**
Feijen-de Jong	2011	**4**	**3**	**3**	**5**	**2**

Firdous	2020	**4**	**4**	**4**	**3**	**2**
Glassman	2013	**1**	**3**	**2**	**3**	**2**
Gopalan	2014	**4**	**5**	**4**	**6**	**2**
Higginbottom	2019	**3**	**4**	**5**	**5**	**2**
Hollowell	2011	**3**	**4**	**5**	**4**	**2**
Hunter (a)	2017	**5**	**4**	**5**	**6**	**2**
Hunter (b)	2017	**5**	**4**	**5**	**6**	**2**
Jhaveri	2021	**2**	**5**	**0**	**3**	**2**
Kirubarajan	2022	**4**	**5**	**4**	**5**	**2**
Knight	2013	**3**	**5**	**3**	**3**	**2**
Kyei-Nimakoh	2017	**5**	**4**	**3**	**4**	**2**
Lagarde	2007	**4**	**5**	**4**	**5**	**2**
Lagarde	2008	**5**	**5**	**4**	**6**	**3**
Lassi	2012	**5**	**5**	**5**	**6**	**2**
Lassi	2016	**5**	**5**	**5**	**6**	**3**
Lee	2009	**4**	**4**	**3**	**4**	**2**
Lewin	2010	**5**	**5**	**5**	**6**	**3**
Lyall	2021	**5**	**4**	**5**	**6**	**2**
Magwood	2018	**4**	**4**	**5**	**6**	**2**
Magwood	2019	**5**	**5**	**5**	**5**	**3**
Malqvist	2012	**4**	**3**	**1**	**3**	**2**
Malqvist	2013	**4**	**5**	**5**	**3**	**2**
McArthur	2013	**5**	**5**	**3**	**5**	**2**
McCollum	2016	**5**	**5**	**5**	**5**	**3**
Mengist	2021	**5**	**5**	**5**	**6**	**3**
Moncrieff	2021	**5**	**5**	**4**	**5**	**2**
Muralidharan	2015	**4**	**4**	**3**	**3**	**2**
Murray	2012	**4**	**4**	**5**	**3**	**2**
Mutambudzi	2017	**4**	**1**	**2**	**3**	**2**
Ogundele	2020	**3**	**5**	**4**	**5**	**2**

Palmer	2020	**5**	**5**	**5**	**6**	**3**
Prady	2019	**5**	**3**	**4**	**5**	**3**
Rowe	2003	**3**	**5**	**2**	**3**	**2**
Rowe	2004	**3**	**5**	**3**	**3**	**2**
Sanogo	2019	**4**	**4**	**5**	**6**	**3**
Say	2007	**4**	**5**	**4**	**4**	**1**
Sidze	2021	**4**	**4**	**3**	**6**	**2**
Small	2014	**4**	**3**	**2**	**3**	**2**
Toh	2022	**5**	**4**	**3**	**6**	**2**
Tokhi	2018	**5**	**4**	**5**	**6**	**3**
Vanstone	2019	**2**	**3**	**1**	**2**	**2**
Victoria	2012	**4**	**2**	**1**	**4**	**2**
Walker	2019	**5**	**4**	**4**	**5**	**2**
Watson	2019	**5**	**5**	**5**	**4**	**2**
Wekesah	2016	**4**	**5**	**3**	**5**	**2**
Widmer	2011	**4**	**5**	**2**	**3**	**2**
Yuan	2012	**4**	**3**	**2**	**5**	**2**
Yuan	2014	**4**	**5**	**4**	**4**	**2**
