# Supplementary material for: Equity in prenatal healthcare services globally: an umbrella review
Source: BMC Pregnancy Childbirth. 2024 Mar 11;24:191. doi: 10.1186/s12884-024-06388-0 (PMC10926563; doi:10.1186/s12884-024-06388-0)
Supplement: Supplementary file 4 — Additional file 4. List of included studies and study characteristics. Complete list of included studies in umbrella review and study characteristics (author, year of publication, title, aim, countries of studies analyzed within included reviews, population, health services focus, outcomes, funding). [file 12884_2024_6388_MOESM4_ESM.pdf]

Characteristics of Included Studies

First Author Last Name	Publication Year	First Author Country	Title	Aim	Countries of studies analyzed within included reviews	Population Focus	Health Services Focus	Outcomes Relevant to PROGRESS-Plus	Funding Source Reported
Rowe	2003	UK	Social class, ethnicity and attendance for antenatal care in the United Kingdom: a systematic review	To address whether there are social class/ethnic inequalities in attendance of prenatal care in the United Kingdom for pregnant women.	UK	Prenatal only	Prenatal Testing/ Screening	Occupation, Race, Ethnicity, Culture, Language	Yes
Rowe	2004	UK	Social and ethnic inequalities in the offer and uptake of prenatal screening and diagnosis in the UK: a systematic review	To address whether there are social inequalities in the offer/uptake of prenatal testing in the United Kingdom for pregnant women.	UK	Prenatal only	Prenatal Testing/ Screening	Socioeconomic Status, Race, Ethnicity, Culture, Language	Yes
Lagarde	2007	UK	Conditional Cash Transfers for Improving Uptake of Health Interventions in Low- and Middle- Income Countries	To assess the effectiveness of conditional cash transfers in improving access to and use of health services and improving health outcomes, in low- and middle-income countries for women and children.	Mexico, Nicaragua, Malawi, Honduras, Brazil, Colombia	Maternal & Child Health	Demand Side Financing	Socioeconomic Status	Yes

First Author Last Name	Publication Year	First Author Country	Title	Aim	Countries of studies analyzed within included reviews	Population Focus	Health Services Focus	Outcomes Relevant to PROGRESS-Plus	Funding Source Reported
Say	2007	Switzerland	A systematic review of inequalities in the use of maternal health care in developing countries: examining the scale of the problem and the importance of context	To assess the evidence of key maternal healthcare interventions in developing countries by women's place of residence and socioeconomic status for pregnant women aged 15-49 years.	Turkey, Tajikistan, Nepal, Kenya, Ethiopia, India, Paraguay, Uganda, United Republic of Tanzania, China, Bangladesh, Lao People's Democratic Republic, Ghana, Jamaica, Morocco, Belize, Viet Nam, Guatemala, Zimbabwe, Botswana, South Africa, Brazil, Ecuador	Prenatal only	General	Place of Residence, Race, Ethnicity, Culture, Language, Socioeconomic Status	Yes
Lagarde	2008	UK	The impact of user fees on health service utilization in low- and middle-income countries: how strong is the evidence?	To assess the effect on health service utilization of user fees in low- and middle-income countries.	Burkina Faso, Kenya, Papau New Guinea, Niger, Cameroon, Uganda, South Africa, Columbia, Sudan, Lesotho, Gabon, Ecuador	General	User Fees	Socioeconomic Status	Yes
Lee	2009	USA South Africa	Linking families and facilities for care at birth: What works to avert intrapartum-related deaths?	To describe the evidence for interventions to link mothers with skilled care during pregnancy, labor, and birth for	Bolivia, Nepal, Bangladesh, Pakistan, India, Burkino Faso, South Africa, Ghana, Nigeria, Guatemala,	Maternal Health	General	Socioeconomic Status, Race, Ethnicity, Culture, Language, Social Capital,	Not Reported

First Author Last Name	Publication Year	First Author Country	Title	Aim	Countries of studies analyzed within included reviews	Population Focus	Health Services Focus	Outcomes Relevant to PROGRESS-Plus	Funding Source Reported
				mothers in low- and middle-income settings.	Gambia, DR of Congo, Kenya, Tanzania, Rwanda, Mauritania, Senegal, Mali, Honduras, Mexico, India, Cambodia, Sierra Leone, Malawi, Uganda, Ethiopia, Zimbabwe, Cuba,			Place of Residence	
Darmstadt	2009	USA	60 million non-facility births: Who can deliver in community settings to reduce intrapartum-related deaths?	To review the evidence for the effect of care by different community groups during pregnancy and childbirth for pregnant women and neonates in low- middle-income settings.	Nepal, Vietnam, Indonesia, England, Zambia, Bangladesh, Benin, Ecuador, Jamaica, Rwanda, Nicaragua, China, Sudan, Malaysia, Mozambique, Sweden, Ghana, India, Guatemala, Gambia, Argentina, DR Congo, Guatamala, Pakistan, Zambia	Maternal & Child Health	Community Organizations	Education	No Funding

First Author Last Name	Publication Year	First Author Country	Title	Aim	Countries of studies analyzed within included reviews	Population Focus	Health Services Focus	Outcomes Relevant to PROGRESS-Plus	Funding Source Reported
Lewin	2010	Norway	Lay health workers in primary and community health care for maternal and child health and the management of infectious diseases (review)	To assess the effects of lay health worker (LHW) interventions in primary and community health care on any population, with an intent to improve maternal and child health and the management of infectious diseases.	Australia, Canada, Ireland, New Zealand, UK, USA, Brazil, China, India, Mexico, Philipines, Thailand, Turkey, South Africa, Bangladesh, Burkina Faso, Ethiopia, Ghana, Iraq, Jamaica, Nepal, Pakistan, Tanzania, Vietnam	General	Lay/Community Health Workers	Education, Socioeconomic Status, Race, Ethnicity, Culture, Language, Place of Residence, Social Capital	Yes
DeMaio	2010	Canada	Immigration as pathogenic: a systematic review of the health of immigrants to Canada	To explore and highlight the state of current knowledge on the health transition effects of immigrants to Canada compared to a Canadian-born population and how this is influenced by ethnicity, race, socioeconomic status, and contextual factors.	Canada	General	General	Race, Ethnicity, Culture, Language, Socioeconomic Status	No Funding

First Author Last Name	Publication Year	First Author Country	Title	Aim	Countries of studies analyzed within included reviews	Population Focus	Health Services Focus	Outcomes Relevant to PROGRESS-Plus	Funding Source Reported
Feijen-de Jong	2011	Netherlands	Determinants of late and/or inadequate use of prenatal healthcare in high-income countries: a systematic review	To review the current evidence of the determinants of use of prenatal healthcare in high-income countries for pregnant women.	USA, Canada, UK, Finland	Prenatal only	General	Race, Ethnicity, Culture, Language, Socioeconomic Status	Yes
Hollowell	2011	UK	The effectiveness of antenatal care programmes to reduce infant mortality and preterm birth in socially disadvantaged and vulnerable women in high-income countries: a systematic review	To identify the best available evidence on the effectiveness of interventions focused on antenatal care to reduce infant mortality in socially disadvantaged groups of women and other specific groups, such as teenagers and substance abusers, with risk factors for adverse birth outcomes strongly asso-ciated with social disadvantage.	USA, Australia, UK, Canada, Greece	Prenatal only	General	Socioeconomic Status, Place of Residence	Yes
Widmer	2011	Switzerland	The role of faith-based organizations in maternal and newborn health care in Africa	To assess the work of faith-based organizations in the area of maternal/newborn healthcare in Africa during the past 2 decades,	Ghana, Malawi, Mozambique, Nigeria, Uganda, and Tanzania, Democratic Republic of Congo	Maternal & Child Health	Faith-based/Community organization	Religion	Not Reported

First Author Last Name	Publication Year	First Author Country	Title	Aim	Countries of studies analyzed within included reviews	Population Focus	Health Services Focus	Outcomes Relevant to PROGRESS-Plus	Funding Source Reported
Victoria	2012	Brazil	Scaling up maternal nutrition programs to improve birth outcomes: A review of implementation issues	To analyze the evidence on impact and cost-effectiveness of nutrition programs for maternal care during pregnancy.	Nicaragua, Nepal, India, Bangladesh, Madagascar, Mexico, Nigeria, South Africa, Brazil	Prenatal only	Nutrition	Socioeconomic Status	Not Reported
Lassi	2012	Pakistan	Community-based intervention packages for reducing maternal morbidity and mortality and improving neonatal outcomes	To assess the effectiveness of community-based interventions in reducing maternal and neonatal morbidity and mortality and improving neonatal outcomes, and the impact of different interventions for pregnant women in underdeveloped countries.	Bangledesh, india, indonesia, nepal, pakistan, burkino faso, gambia, greece, bolivia	Prenatal only	Community Organizations	Social Capital	Yes
Yu	2012	UK	A systematic review of issues around antenatal screening and prenatal diagnostic testing for genetic disorders: women of Asian origin in western countries	To identify, describe and critique literature on antenatal screening and prenatal diagnostic testing for genetic conditions for women of Asian origin in western countries.	UK, Australia, USA, Canada,	Prenatal only	Prenatal Testing/ Screening	Race, Ethnicity, Culture, Language	Not Reported

First Author Last Name	Publication Year	First Author Country	Title	Aim	Countries of studies analyzed within included reviews	Population Focus	Health Services Focus	Outcomes Relevant to PROGRESS-Plus	Funding Source Reported
Murray	2012	United Kingdon	Demand-side financing measures to increase maternal health service utilisation and improve health outcomes: a systematic review of evidence from low- and middle-income countries	To assess the effects of demand-side financing (DSF) interventions on maternal health service utilisation and maternal health outcomes for poor, rural or socially excluded women of all ages who were either pregnant or within 42 days of conclusion of pregnancy in low- and middle-income countries.	Turkey, Bangladesh, Kenya, India, Indonesia, Cambodia, Ghana, Bolivia, Tanzania, Uganda, Nepal, Armenia	Perinatal	Demand Side Financing	Place of Residence, Socioeconomic Status, Social Capital, Race, Ethnicity, Culture, Language	Yes
Malqvist	2012	Sweden	Causes and determinants of inequity in maternal and child health in Vietnam	To promote policy action through mapping the known causes and determinants of inequity in maternal and child health in Vietnam.	N/R	Maternal & Child Health	General	Race, Ethnicity, Culture, Language, Socioeconomic Status, Gender/Sex, Education	Yes
Balaam	2013	UK	A qualitative review of migrant women's perceptions of their needs and experiences related to pregnancy and childbirth	To synthesize evidence of migrant women's (refugees, illegal, asylum-seekers, economic migrants, maternity care experiences, etc.) perceptions of their health needs and experiences in relation to pregnancy and childbirth.	Sweden, UK, Switzerland, Norway, Ireland, Greece.	Perinatal	General	Race, Ethnicity, Culture, Language, Religion, Gender/Sex, Social Capital	Yes

First Author Last Name	Publication Year	First Author Country	Title	Aim	Countries of studies analyzed within included reviews	Population Focus	Health Services Focus	Outcomes Relevant to PROGRESS-Plus	Funding Source Reported
Glassman	2013	USA	Impact of Conditional Cash Transfers on Maternal and Newborn Health	To assess the effect of Conditional Cash Transfers (CCT) on maternal and neonatal health outcomes, the use or provision of maternal health services, or the care-seeking behaviour by women.	El Salvador, Honduras, Nepal, Mexico, Guatemala, Uruguay, India	Maternal & Child Health	Demand Side Financing	Socioeconomic Status	Not Reported
Knight	2013	UK	Why Are Women Dying When They Reach Hospital on Time? A Systematic Review of the 'Third Delay'	To identify and categorise facility-level barriers (phase III delays) to the provision of evidence-based emergency obstetric care in developing countries.	Morocco, Nicaragua, Sri Lanka, Niger, Rwanda, Tanzania, Peru, Pakistan, Vietnam, Bhutan, Cameroon, India, Bolivia, El Savador, Honduras, Gabon, Guinneau-Bissau, Gambia, Benin, Chad	Perinatal	General	Religion, Socioeconomic Status, Race, Ethnicity, Culture, Language	No Funding
Byrne	2013	Australia	Looking Beyond Supply: A Systematic Literature Review of Demand-Side Barriers to Health Service Utilization in the Moutains of Nepal	To identify demand-side barriers to the utilization of formal reproductive, maternal, newborn, or child services in the Mountains ecological region of Nepal (low-middle-income).	Nepal	Maternal & Child Health	General	Education, Race, Ethnicity, Culture, Language, Plus-Age, Gender/Sex	Yes

First Author Last Name	Publication Year	First Author Country	Title	Aim	Countries of studies analyzed within included reviews	Population Focus	Health Services Focus	Outcomes Relevant to PROGRESS-Plus	Funding Source Reported
Chamberlain	2013	Australia	Psychosocial interventions for supporting women to stop smoking in pregnancy (Review)	To assess the effects of smoking cessation interventions during pregnancy on smoking behaviour and perinatal health outcomes.	United States, England, Argentina, Brazil, Cuba, Mexico, New Zealand, Newcastle, New South Wales, Australia, United Kingdom, Columbia, Norway, Netherlands, Sweden, Canada, Poland, Scotland, Ireland, Spain	Perinatal	Smoking Cessation	Social Capital	Yes
McArthur	2013	Australia	Maternal mortality in Cambodia, Thailand, Malaysia and Sri Lanka: a systematic review of local and national policy and practice and initiatives.	To review health system/service delivery structures and policies that have reduced maternal mortality for pregnant and birthing women who received care from a skilled birth attendant within Cambodia, Malaysia, Thailand and Sri Lanka.	Sri Lanka, Cambodia, Thailand, Malaysia	Perinatal	General	Place of Residence, Socioeconomic Status, Gender/Sex, Education	Not Reported
Malqvist	2013	Sweden	Targeted Interventions for Improved Equity in Maternal and Child Health in Low- and	To display the evidence of targeted maternal and child health interventions in their effectiveness to reduce inequity in	Pakistan, Bangladesh, Nicaragua, Mexico, Cambodia, India, Honduras, Peru, Kenya	Maternal & Child Health	General	Socioeconomic Status	Yes

First Author Last Name	Publication Year	First Author Country	Title	Aim	Countries of studies analyzed within included reviews	Population Focus	Health Services Focus	Outcomes Relevant to PROGRESS-Plus	Funding Source Reported
			Middle-Income Settings: A Systematic Review and Meta-Analysis	low and middle-income countries.					
Ehiri	2014	USA	Training and deployment of lay refugee/internally displaced persons to provide basic health services in camps: a systematic review	To assess the effects of interventions that train and deploy lay refugees/internally displaced women (≥15 years) to provide basic health service to maternal and child populations in refugee/internally displaced camps.	Guinea, Belize, Myanmar, Thai-Myanmar Border, Tanzania	Maternal & Child Health	Lay/Community Health Workers	Social Capital	No Funding
Benova	2014	UK	Socio-Economic Gradients in Maternal and Child Health-Seeking Behaviours in Egypt: Systematic Literature Review and Evidence Synthesis	To identify and synthesize evidence about the existence, magnitude and trends in socio-economic gradients in health-seeking behaviours related to maternal and child health in Egypt.	Egypt	Maternal & Child Health	General	Socioeconomic Status, Education	Yes
Small	2014	Australia	Immigrant and non-immigrant women's experiences of maternity care: a systematic and comparative	To compare immigrant (women not born in the country they are giving birth) and non-immigrant women's	Australia, Canada, Sweden, United Kingdom, USA	Perinatal	General	Race, Ethnicity, Culture, Language	Not Reported

First Author Last Name	Publication Year	First Author Country	Title	Aim	Countries of studies analyzed within included reviews	Population Focus	Health Services Focus	Outcomes Relevant to PROGRESS-Plus	Funding Source Reported
			review of studies in five countries	experiences and needs in maternity care, within and across countries.					
Byrne	2014	Australia	What Works? Strategies to Increase Reproductive, Maternal and Child Health in Difficult to Access Mountainous Locations: A Systematic Literature Review	To identify strategies to address barriers to care-seeking, acceptability, satisfaction, and/or utilization of reproductive, maternal, newborn, and child service in low- and lower-middle income countries, in difficult-to-reach mountainous regions.	Afghanistan, Bolivia, Ethiopia, Guatemala, Indonesia, Kenya, Kyrgyzstan, Nepal, Pakistan, Papua New Guinea and Tajikistan.	Maternal & Child Health	General	Socioeconomic Status, Place of Residence, Social Capital	Yes
Gopalan	2014	UK	Health sector demand-side financial incentives in low- and middle-income countries: A systematic review on demand- and supply-side effects	To investigate the demand- and supply-side effects of consumer-led financial incentives in low- and middle-income countries	Mexico, Honduras, Brazil, Columbia, Nicaragua, Chile, Turkey, Jamaica, Latin America, Mexico, Ecuador, Malawi, China, India, Tanzania, Nepal, Bangladesh, Madagascar.	General	Demand Side Financing	Socioeconomic Status, Place of Residence	Not Reported

First Author Last Name	Publication Year	First Author Country	Title	Aim	Countries of studies analyzed within included reviews	Population Focus	Health Services Focus	Outcomes Relevant to PROGRESS-Plus	Funding Source Reported
Yuan	2014	Sweden China	What interventions are effective on reducing inequalities in maternal and child health in low- and middle-income settings? A systematic review	To collect evidence about the differential effects of interventions on different sociodemographic groups in order to identify interventions that were effective in reducing maternal or child health inequalities in low-middle-income countries.	Brazil, India, Guinea-Bissau, Bangladesh, Nepal, South Africa, Indonesia, Nigeria, Uganda, Philipines, Tanzania, Indiana, Zambia, China, Colombia.	Maternal & Child Health	General	Socioeconomic Status, Social Capital	Yes
Muralidharan	2015	India	Transforming Gender Norms, Roles, and Power Dynamics for Better Health Evidence from a Systematic Review of Gender-integrated Health Programs in Low-and Middle-Income Countries	To identify and assess gender-accommodating and gender-transformative strategies in health programs in low- middle-income countries and understand their impact on reproductive, maternal, neonatal, child, and adolescent health health outcomes.	India, Bangladesh, Nepal, Afghanistan, Pakistan, Bhutan, Sri Lanka, Maldives	General	Gender/Role-Accomodating & Transformative Strategies	Gender/Sex, Social Capital	Yes
Caliskan	2015	Turkey	Equity in maternal health care service utilization: a systematic review for developing countries	To explore progress of equity in the utilization of maternal health care services in developing countries for	Bangladesh, Burkina Faso, Cambodia, China, Egypt, Ethiopia, India, Kenya, Namibia, Nigeria,	Perinatal	General	Socioeconomic Status, Education	Not Reported

First Author Last Name	Publication Year	First Author Country	Title	Aim	Countries of studies analyzed within included reviews	Population Focus	Health Services Focus	Outcomes Relevant to PROGRESS-Plus	Funding Source Reported
				pregnant or postpartum women.	Pakistan, Republic of Vanuatu, Tajikistan, Thailand, Uganda, Vietnam				
McCollum	2016	UK	How equitable are community health worker programmes and which programme features influence equity of community health worker services? A systematic review	To determine the extent of equity of community health worker programmes and to identify intervention designs which influence equity of health outcomes.	Brazil, Bangladesh, Philippines, Malawi, Kenya, Pakistan, Guatemala, Zambia, Cambodia, Uganda, South Africa, Nigeria, Haiti, Nepal, Tanzania,	General	Lay/Community Health Workers	Place of Residence, Socioeconomic Status, Social Capital, Education, Race, Ethnicity, Culture, Language	Yes
Lassi	2016	Australia	Strategies for improving health care seeking for maternal and newborn illnesses in low- and middle-income countries: a systematic review and meta-analysis	To assess the impact of different strategies to improve maternal and neonatal health care seeking in low- and middle- income countries for pregnant women at any gestation, postpartum women up to 6 weeks after giving birth, and neonates less than 28 days of life.	India, Bangladesh, Vietnam, Malawi, Ghana, South Africa, Tanzania, Pakistan, China, Zambia, Nigeria, Syria, Uganda, Nepal, Gambia, Eritrea, Indonesia, Burkino Faso, Brazil, Guatemala, Thailand, Bolivia, Argentina,	Maternal & Child Health	General	Place of Residence, Social Capital, Education	Yes

First Author Last Name	Publication Year	First Author Country	Title	Aim	Countries of studies analyzed within included reviews	Population Focus	Health Services Focus	Outcomes Relevant to PROGRESS-Plus	Funding Source Reported
					Congo DR, Turkey,				
Wekesah	2016	Kenya	Effective non-drug interventions for improving outcomes and quality of maternal health care in sub-Saharan Africa: a systematic review	To explore non-drug interventions and their effectiveness to improve outcomes and impact on the quality of maternal health care in Sub-Saharan Africa for pregnant and postpartum women.	Rwanda, Tanzania, Nigeria, South Africa, Ghana, Kenya, Mali, Burkina Faso, Nairobi, Senegal, Burundi, Uganda, Malawi, Angola, Somali, Ethiopia, Mozambique, Zambia, Benin, Côte d'Ivoire	Perinatal	General	Place of Residence, Socioeconomic Status, Social Capital, Education	Yes
Ahmed	2017	Canada	A mixed methods systematic review of success factors of mhealth and telehealth for maternal health in Sub-Saharan Africa	To provide a knowledge synthesis regarding success factors of introduction and use of telehealth and mhealth for maternal health in Sub-Saharan Africa for women of childbearing age (15-50 years).	Ethiopia, Rwanda, Ghana, Liberia, South Africa, Kenya,	Maternal Health	Digital/Mhealth/Telehealth	Race, Ethnicity, Culture, Language, Social Capital	Not Reported

First Author Last Name	Publication Year	First Author Country	Title	Aim	Countries of studies analyzed within included reviews	Population Focus	Health Services Focus	Outcomes Relevant to PROGRESS-Plus	Funding Source Reported
Hunter b	2017	UK	Demand-side financing for maternal and newborn health: what do we know about factors that affect implementation of cash transfers and voucher programmes?	To document experiences and implementation factors associated with use of demand-side financing to promote uptake of maternity care services and maternal and newborn health, and wider impact on quality of care for women who were pregnant or within 42 days of end of pregnancy, in low- and middle-income countries.	Mexico, Indonesia, Brazil, Salvador, India, Uruguay, Guatemala, Honduras, Zambia, Nigeria, China, Nepal, Pakistan, Bangladesh, Uganda, Kenya, Cambodia, Tanzania	Perinatal	Demand Side Financing	Place of Residence, Socioeconomic Status, Race, Ethnicity, Culture, Language, Gender/Sex	Yes
Mutambudzi	2017	USA	A review of recent literature on materialist and psychosocial models for racial and ethnic disparities in birth outcomes in the US, 2000–2014	To explore the implications of materialistic and psychosocial models in racial and ethnic disparities of birth outcomes, and for reforming both the work environment and the cycle of poverty for singleton, pregnant women, black women and other racial ethnic groups in the United States.	USA	Prenatal only	General	Race, Ethnicity, Culture, Language, Socioeconomic Status	Not Reported

First Author Last Name	Publication Year	First Author Country	Title	Aim	Countries of studies analyzed within included reviews	Population Focus	Health Services Focus	Outcomes Relevant to PROGRESS-Plus	Funding Source Reported
Kyei-Nimakoh	2017	Australia	Access barriers to obstetric care at health facilities in sub-Saharan Africa—a systematic review	To synthesize current evidence on barriers to obstetric care at health institutions in Sub-Saharan Africa for females in their reproductive age, pregnant and postnatal women.	Nigeria, Ethiopia, Uganda, Nigeria, Zimbabwe, Burkina Faso, Tanzania, Sierra Leone, Rwanda, rural Malawi, Kenya, Ghana, Zambia, South Africa, Cameroon, Mali, Democratic Republic of Congo, Mozambique, Burundi and Northern Uganda, Senegal, Congo, Benin, Gambia, Angola, Liberia,	Perinatal	General	Race, Ethnicity, Culture, Language, Religion, Education, Social Capital, Place of Residence	Yes
Hunter a	2017	UK	The effects of cash transfers and vouchers on the use and quality of maternity care services: A systematic review	To assess the effect and cost-effectiveness of different demand-side financing interventions on maternity care service utilisation and maternal and newborn health outcomes in low- and middle-income countries for women who were pregnant or within	Pakistan, Bangladesh, Indonesia, Uganda, Uruguay, Kenya, india, Mexico, El Salvador, Brazil, Guatemala, Zambia, Tanzania, China, Honduras, Tanzania,	Perinatal	Demand Side Financing	Socioeconomic Status	Yes

First Author Last Name	Publication Year	First Author Country	Title	Aim	Countries of studies analyzed within included reviews	Population Focus	Health Services Focus	Outcomes Relevant to PROGRESS-Plus	Funding Source Reported
				42 days of giving birth.	Nigeria, Nepal, Cambodia				
Cormick	2018	Argentina	Global inequities in dietary calcium intake during pregnancy: a systematic review and meta-analysis and relevant stakeholders, through the following objectives: investigate the experience of provision of	To assess calcium intake anytime during pregnancy for pregnant women of any age, ethnic group, parity, education, and socio-economic status.	Australia, Belgium, Canada, Croatia, Greece, Ireland, Israel, Japan, Netherlands, New Zealand, Norway, Poland, Portugal, Saudi Arabia, South Korea, Spain, Taiwan, UK, USA, Argentina, Brazil, China, Colombia, Egypt, Ethiopia, India, Jordan, Kenya, Malaysia, Morocco, Nepal, Pakistan, South Africa, Thailand, Turkey	Prenatal only	Nutrition	Socioeconomic Status	Yes

First Author Last Name	Publication Year	First Author Country	Title	Aim	Countries of studies analyzed within included reviews	Population Focus	Health Services Focus	Outcomes Relevant to PROGRESS-Plus	Funding Source Reported
Tokhi	2018	Australia	Involving men to improve maternal and newborn health: A systematic review of the effectiveness of interventions	To determine the effect of interventions to engage men during pregnancy, childbirth and infancy and their influence on mortality and morbidity in low- and middle-income countries.	South Africa, Pakistan, Nepal, Turkey, India, Bangladesh, Tanzania, Indonesia, Eritrea	Maternal & Child Health	Engaging Men During Pregnancy	Social Capital, Education	Yes
Magwood	2018	Canada	Understanding women's, caregivers', and providers' experiences with home-based records: A systematic review of qualitative studies	To explore the feasibility, acceptability, affordability and equity of home-based records for maternal, newborn and child health in low-, middle-, and high-income settings.	USA, New Zealand, Brazil, Palestine, Australia, South Africa, Scotland, England, Canada, Cambodi	Maternal & Child Health	Home-based Records	Education	Yes
Blanchard	2019	UK	Effects of community health worker interventions on socioeconomic inequities in maternal and newborn health in low-income and middle-income countries: a mixed-methods systematic review	To explore the effects of community health worker interventions on equity in maternal and newborn health in low- middle-income countries.	India, Malawi, Bangaldesh, Nepal, Vietnam, Brazil	Maternal & Child Health	Lay/Community Health Workers	Socioeconomic Status, Social Capital, Place of Residence, Religion	Yes

First Author Last Name	Publication Year	First Author Country	Title	Aim	Countries of studies analyzed within included reviews	Population Focus	Health Services Focus	Outcomes Relevant to PROGRESS-Plus	Funding Source Reported
Walker	2019	New Zealand	Understanding the experiences, perspectives and values of indigenous women around smoking cessation in pregnancy: systematic review and thematic synthesis of qualitative studies	To describe indigenous women's experiences, perceptions, and values related to stopping smoking in pregnancy.	New Zealand, Australia	Prenatal only	Smoking Cessation	Race, Ethnicity, Culture, Language, Social Capital, Place of Residence, Socioeconomic Status	No Funding
Higgenbottom	2019	UK	Experience of and access to maternity care in the UK by immigrant women: a narrative synthesis systematic review	To explore interventions that focus specifically on improving maternity care for immigrant women in the United Kingdom and how they address inequality, accessibility and acceptability.	UK	Perinatal	General	Race, Ethnicity, Culture, Language, Socioeconomic Status, Place of Residence, Religion	Yes
Magwood	2019	Canada	Effectiveness of home-based records on maternal, newborn and child health outcomes: A systematic review and meta-analysis	To synthesize and compare the evidence of the health and cost effectiveness of home based records for improving maternal, newborn and child health out- comes.	Norway, England, Australia, Mongolia, Indonesia, Canada, USA, Pakistan, Cambodia	Maternal & Child Health	Home-based Records	Education	Yes

First Author Last Name	Publication Year	First Author Country	Title	Aim	Countries of studies analyzed within included reviews	Population Focus	Health Services Focus	Outcomes Relevant to PROGRESS-Plus	Funding Source Reported
Banke-Thomas	2019	UK	Assessing geographical distribution and accessibility of emergency obstetric care in sub-Saharan Africa: a systematic review	To explore studies that assessed emergency obstetric care geographical distribution and accessibility in Sub-Saharan Africa for women during pregnancy and childbirth.	Malawi, Ethiopia, Zambia, Sierra Leone, Ghana, Rwanda, Kenya, Burkina Faso, Guinea, Tanzania	Perinatal	Emergency Obstetric Care	Place of Residence	No Funding
Ames	2019	Norway	Clients' perceptions and experiences of targeted digital communication accessible via mobile devices for reproductive, maternal, newborn, child, and adolescent health: a qualitative evidence synthesis (Review)	To explore clients' perceptions and experiences of targeted digital communication via mobile devices on topics related to reproductive, maternal, newborn, child, or adolescent health.	Nigeria, USA, Peru, Ghana, UK, Austraila, Lesotho, Sierra Leone, Kenya, South Africa, Cameroon, India, Canada, Uganda, Cambodia,	Maternal & Child Health	Digital/Mhealth/Telehealth	Education, Socioeconomic Status, Race, Ethnicity, Culture, Language	Yes
Bovill	2019	Australia	Ngu-ng-gi-la-nha (to exchange) knowledge. How is Aboriginal and Torres Strait Islander people's empowerment being upheld and reportedin	To provide an overview of smoking cessation interventions offered to Aboriginal women during the pregnancy or postnatal period and describe the evidence of individual and	Australia	Perinatal	Smoking Cessation	Social Capital, Education	Yes

First Author Last Name	Publication Year	First Author Country	Title	Aim	Countries of studies analyzed within included reviews	Population Focus	Health Services Focus	Outcomes Relevant to PROGRESS-Plus	Funding Source Reported
			smoking cessation interventions during pregnancy: a systematic review	community empowerment in Australia.					
Watson	2019	UK	A systematic review of ethnic minority women's experiences of perinatal mental health conditions and services in Europe	To explore the experiences and perceptions of perinatal mental health and help-seeking behaviours for mental health services amongst ethnic minority women in Europe.	UK	Perinatal	Mental Health	Race, Ethnicity, Culture, Language, Gender/Sex	Yes
Vanstone	2019	Canada	Perspectives of Pregnant People and Clinicians on Noninvasive Prenatal Testing: A systematic Review and Qualitative Meta-synthesis	To describe belief preferences, and perspectives of pregnant people, their families, clinicians, and others who have experience with noninvasive prenatal testing.	US, UK, Netherlands, Canada, China, Finaldn, Israel, New Zealand	Prenatal only	Prenatal Testing/ Screening	Socioeconomic Status	Yes
Sanogo	2019	Canada	Universal Health Coverage and Facilitation of Equitable Access to Care in Africa	To examine the effects of universal health coverage on equitable access to care in Africa for vulnerable and underpriveleged populations.	Ghana, Brazil, Mexico, Thailand, Malawi, Madagascar, Burkina Faso, Rwanda, Bangladesh, Vietnam, Kenya	General	Universal Health Coverage	Socioeconomic Status	Not Reported

First Author Last Name	Publication Year	First Author Country	Title	Aim	Countries of studies analyzed within included reviews	Population Focus	Health Services Focus	Outcomes Relevant to PROGRESS-Plus	Funding Source Reported
Fair	2020	UK	Migrant women's experiences of pregnancy, childbirth and maternity care in European countries: A systematic review	To provide evidence on migrant women's experiences of pregnancy, childbirth and maternity care in their destination country within Europe for women described as migrants, refugees or asylum seekers, including undocumented migrants.	Portugal, UK, Spain, Switzerland, Sweden, Finland, Ireland, Norway, Netherlands, Greece, Germany, France, Italy, Czech Republic	Perinatal	General	Race, Ethnicity, Culture, Language, Socioeconomic Status, Occupation, Religion	Yes
Firdous	2020	UK	Muslim women's experiences of maternity services in the UK: qualitative systematic review and thematic synthesis	To identify and synthesize evidence of Muslim women's experiences of maternity services in the United Kingdom.	United Kingdom	Perinatal	General	Religion, Race, Ethnicity, Culture, Language	Yes
Palmer	2020	UK	Targeted client communication via mobile devices for improving maternal, neonatal, and child health	To assess the effects of targeted client communication via mobile devices on health behaviour, service use, health, and well-being for maternal, newborn, and child health.	USA, Thailand, India, Kenya, Tanzania, Ecudaor, England, Nigeria, Canada, Zimbabwe, Cameroon, Guatemala, Hawaii, India	Maternal & Child Health	Digital/Mhealth/Telehealth	Place of Residence, Social Capital	Yes

First Author Last Name	Publication Year	First Author Country	Title	Aim	Countries of studies analyzed within included reviews	Population Focus	Health Services Focus	Outcomes Relevant to PROGRESS-Plus	Funding Source Reported
Dahab	2020	UK	Barriers to Accessing Maternal Care in Low Income Countries in Africa: A Systematic Review	To investigate the experience of provision of maternal care services and facilities in low-income countries in Africa and explore the different barriers to accessing maternal care reported by women and relevant stakeholders during pregnancy, delivery, and the immediate period after childbirth.	South Sudan, Ethiopia, Mali, Togo, Mozambique, Malawi,Tanzania, Zimbabwe	Perinatal	General	Socioeconomic Status, Place of Residence, Race, Ethnicity, Culture, Language, Occupation, Social Capital	No Funding
Ogundele	2020	Netherlands	Socioeconomic inequalities in reproductive health care services across Sub-Saharan Africa. A systematic review and meta-analysis Author links open overlay panel	To analyze evidence on socioeconomic inequalities in reproductive health care utilization in Sub-Saharan Africa for women of reproductive age	Ghana, Nigeria, South Africa, Cameroon, Ethiopia, Ghana, Kenya, Nigeria, Uganda, Zambia, Zimbabwe, Malawi, Burkina, Faso, Namibia	Perinatal	General	Socioeconomic Status	Not Reported
Beatson	2020	Australia	Systematic review: An exploration of core componentry characterizing effective sustained nurse	To explore program components aspects that nursing home visiting programs have in common and the program content which	United Kingdom, Australia, USA, Germany, Netherlands	Perinatal	Nursing Home Visit Programs	Education, Place of Residence, Social Capital	Yes

First Author Last Name	Publication Year	First Author Country	Title	Aim	Countries of studies analyzed within included reviews	Population Focus	Health Services Focus	Outcomes Relevant to PROGRESS-Plus	Funding Source Reported
			home visiting programs	accounts for their effectiveness on relevant outcomes for pregnant women, disadvantaged mothers, during pregnancy or prior to the child's first birthday.					
Chando	2021	Australia	Stakeholder perspectives on the implementation and impact of Indigenous health interventions: A systematic review of qualitative studies	To describe perspectives and experiences with the implementation and impact of complex health interventions for Indigenous communities.	United States, Canada, Australia, New Zealand	General	General	Race, Ethnicity, Culture, Language, Place of Residence, Social Capital	Yes
Mengist	2021	Ethiopia	Maternal near miss in Ethiopia: Protective role of antenatal care and dispartiy in socioeconomic inequities: A systematic review and meta-analysis.	To estimate the national burden of maternal near-miss and contributing factors in Ethiopia for women who were pregnant, in childbirth, or postpartum.	Ethiopia	Perinatal	General	Place of Residence, Education, Social Capital	No Funding
Moncrieff	2021	UK	First and second trimester ultrasound in pregnancy: A systematic review and	To examine what views, beliefs, concerns and experiences have been reported in relation to routine	Sweden, UK, Canada, Denmark, USA, Brazil, Norway, Tanzania, Greece, Kenya,	Prenatal only	Prenatal Testing/ Screening	Socioeconomic Status, Place of Residence	Yes

First Author Last Name	Publication Year	First Author Country	Title	Aim	Countries of studies analyzed within included reviews	Population Focus	Health Services Focus	Outcomes Relevant to PROGRESS-Plus	Funding Source Reported
			metasynthesis of the views and experiences of pregnant women, partners, and health workers	antenatal ultrasound examination in pregnancy and what are the influencing factors associated with appropriate or inappropriate use of routine antenatal ultrasounds?	Australia, Vietnam, Holland, India, Israel, New Zealand, Iceland, Uganda, Netherlands				
Lyall	2021	Australia	"The Problem Is that We Hear a Bit of Everything . . . ": A Qualitative Systematic Review of Factors Associated with Alcohol Use, Reduction, and Abstinence in Pregnancy	To understand the complexities of women's alcohol use, reduction, or abstinence during pregnancy for pregnant and recently postpartum women.	Australia, USA, UK, South Africa, Switzerland, Brazil, France, India, Netherlands, Sweden	Perinatal	Alcohol Abstinence		Yes
Jhaveri	2021	USA	Responsible Inclusion of Pregnant Individuals in Eradicating HCV	To assess data on hepatitis C virus treatment in pregnancy and analyze ethical considerations for the responsible inclusion of pregnant individuals.	N/R	Prenatal only	Hepatitis C Treatment	Socioeconomic Status	Yes
Sidze	2021	Kenya	Inequalities in Access and Utilization of Maternal, Newborn and Child Health Services in sub-	To explore the state of inequalities in access to and utilization of maternal, newborn and child health services in the sub-	Kenya, Uganda, Ethiopia, Nigeria, Malawi, Tanzania, Zambia, Ghana,	Maternal & Child Health	General	Race, Ethnicity, Culture, Language, Socioeconomic Status, Social Capital	Not Reported

First Author Last Name	Publication Year	First Author Country	Title	Aim	Countries of studies analyzed within included reviews	Population Focus	Health Services Focus	Outcomes Relevant to PROGRESS-Plus	Funding Source Reported
			Saharan Africa: A Special Focus on Urban Settings	Saharan African region.	Burkina Faso, South Sudan				
Prady	2021	UK	Inequalities in the identification and management of common mental disorders in the perinatal period: An equity focused reanalysis of a systematic review	To explore the equity in the implementation of guidance related to identification and management for mental health problems in the United Kingdom for perinatal women.	United Kingdom	Perinatal	Mental Health	Race, Ethnicity, Culture, Language, Socioeconomic Status, Gender/Sex, Plus-Age	Yes
Toh	2022	Singapore	Experiences and needs of women from ethnic minorities in maternity healthcare: A qualitative systematic review and meta-aggregation	To consolidate the available evidence around ethnic minority women's experiences and needs when accessing maternity care.	UK, USA, Ireland, Vietnam, London, New Zealand, Netherlands, England, Balkans, Belgium, Iran, Scotland, Australia, Israel, Alberta	Perinatal	General	Race, Ethnicity, Culture, Language, Gender/Sex	Yes
Kirubarajan	2022	Canada	LGBTQ2S+ childbearing individuals and perinatal mental health: A systematic review	To characterize and synthesize the experiences of LGBTQ2S+ child-bearing individuals regarding perinatal mental health.	Canada, USA, Netherlands, Sweden, Australia,	Perinatal	Mental Health	Gender/Sex, Race, Ethnicity, Culture, Language, Social Capital, Socioeconomic	No Funding

First Author Last Name	Publication Year	First Author Country	Title	Aim	Countries of studies analyzed within included reviews	Population Focus	Health Services Focus	Outcomes Relevant to PROGRESS-Plus	Funding Source Reported
								Status, Place of Residence	

N/R=study did not report this data
