# Supplementary material for: Equity in prenatal healthcare services globally: an umbrella review
Source: BMC Pregnancy Childbirth. 2024 Mar 11;24:191. doi: 10.1186/s12884-024-06388-0 (PMC10926563; doi:10.1186/s12884-024-06388-0)
Supplement: Supplementary file 5 — Additional file 5. List of excluded studies and reason for exclusion. Complete list of excluded studies during search and screening process with reason for exclusion. [file 12884_2024_6388_MOESM5_ESM.pdf]

List of excluded studies and reason for exclusion

**Reason for Exclusion: Not in English language**		
**First Author Last Name**	**Year of Publication**	**Article Title**
Masella	2020	Ethical issues of Internet use by pregnant women during their medical care
Sharifi	2018	The relationship of the structural and intermediate social determinants of health with low birth weight in Iran: A systematic review and meta-analysis
**Reason for Exclusion: Not a systematic review**		
**First Author Last Name**	**Year of Publication**	**Article Title**
Adjei	2019	Factors Influencing Uptake of Institutional Delivery Service by Skilled Birth Attendant's in Ghana: A Framework Analysis of Existing Literature
Albrecht	1994	Assessing the importance of family structure in understanding birth outcomes
Allen	2008	PSHE education on infant feeding: influencing young people's views
Allina	2009	New directions in reproductive health and justice advocacy: yes we can!
Penman-Aguilar	2013	Socioeconomic Disadvantage as a Social Determinant of Teen Childbearing in the U.S
Andermann	2008	Revisiting Wilson and Jungner in the genomic age: a review of screening criteria over the past 40 years
Anderson	2018	Great Expectations: How Gendered expectations shape early mothering experiences
Aston	2014	Universal and targeted early home visiting: perspectives of public health nurses, managers and mothers
Azzopardi-Muscat	2019	Towards an equitable digital public health era: promoting equity through a health literacy perspective
Baily	2008	Ethics, evidence, and cost in newborn screening
Baldwin-Ragaven	2000	Learning from our apartheid past: Human rights challenges for health professionals in contemporary South Africa
Belkengren	2002	Pediatric management problems
Benhalima	2016	Screening for gestational diabetes in Europe: Where do we stand and how to move forward?: A scientific paper commissioned by the European Board & College of Obstetrics and Gynaecology (EBCOG)
Berger	2000	Prenatal cocaine exposure: Long-run effects and policy implications
Bromley	2020	An imaging approach to early pregnancy failure
Broom	2008	Hazardous good intentions? Unintended consequences of the project of prevention
Brown	2019	Redefining postpartum care
Bustreo	2010	Women's and children's health: from pledges to action
Cambronero-Saiz	2013	Gender policies and advertising and marketing practices that affect women's health
Campbell	2021	Pregnancy-Associated Deaths from Homicide, Suicide, and Drug Overdose: Review of Research and the Intersection with Intimate Partner Violence
Caprara	2016	Zika: exposing anew the need for health promotion in Latin America
Carlsen	2014	Maternal education and risk of offspring death; changing patterns from 16 weeks of gestation until one year after birth

Chamberlain	2019	Perinatal opportunities for addressingcomplex intergenerational trauma inAboriginal and Torres Strait Islandercommunities
Chandler	2020	Promoting Optimal Sexual and Reproductive Health with Mobile Health Tools for Black Women: Combining Technology, Culture and Context
Chol	2018	Health system reforms in five sub-Saharan African countries that experienced major armed conflicts (wars) during 1990–2015: a literature review
Chrzan	2008	Social support and nutrition during adolescent pregnancy: Effects on health outcomes of baby and mother
Coleman	2015	Diagnosis of Fetal Anomaly and the Increased Maternal Psychological Toll Associated with Pregnancy Termination
Comrie-Thomson	2015	Challenging gender inequity through male involvement in maternal and newborn health: Critical assessment of an emerging evidence base
Connor	2020	Health risks and outcomes that disproportionately affect women during the Covid-19 pandemic: A review
Correa-De-Araujo	2021	Clinical Outcomes in High-Risk Pregnancies Due to Advanced Maternal Age
Crear-Perry	2021	Social and Structural Determinants of Health Inequities in Maternal Health
Dhami	2009	Ethnicity and access to healthcare
Dixon	2008	Rh(D) immunoglobulin -- what is the evidence for routine prophylaxis during pregnancy
Dixon	2019	Non-invasive pre-natal screening (NIPS) in New Zealand
Dondorp	2015	Non-invasive prenatal testing for aneuploidy and beyond: Challenges of responsible innovation in prenatal screening
Donnellan-Fernandez	2018	Cost-effectiveness of continuity of midwifery care for women with complex pregnancy: a structured review of the literature
Ensor	2004	Overcoming barriers to health service access: influencing the demand side
Esegbona-Adeigbe	2020	COVID-19 and the risk to black, Asian and minority ethnic women during pregnancy
Filippi	2016	Levels and Causes of Maternal Mortality and Morbidity
Fisher	2010	The social determinants of mental health: implications for research and health promotion
Fotso	2015	Leveraging mobile technology to reduce barriers to maternal, newborn and child health care: a contribution to the evidence base
Foulkes	2005	Opportunities for action: addressing Latina sexual and reproductive health
Fowler	2013	Use of maternity surveys in improving the care experience -- a review of the evidence
Garry	2020	Armed conflict and public health: into the 21st century
Gayle	2016	Female genital mutilation and pregnancy: associated risks
George	2018	Gender dynamics in digital health: overcoming blind spots and biases to seize opportunities and responsibilities for transformative health systems
Getz	2001	General practitioners and prenatal testing - follow the experts or scrutinise the issue?
Gottschang	2020	Reproductive Modernities in Policy: Maternal Mortality, Midwives, and Cesarean Sections in China, 1900s–2000s
Graham	2007	Ethics in public health research: minding the gaps: a reassessment of the challenges to safe motherhood

Graham	2010	The public health nursing role in improving outcomes for Mississippi families: recent findings on Mississippi infant mortality
Grant	2020	Sexually Transmitted Infections in Pregnancy: A Narrative Review of the Global Research Gaps, Challenges, and Opportunities
Griffen	2021	Perinatal Mental Health Care In The United States: An Overview Of Policies And Programs
Hall	2014	Assessing the impact of mHealth interventions in low- and middle-income countries – what has been shown to work?
Hammond	2014	Exploring same sex couples' experiences of maternity care
Harper	2021	Health Care Experiences in Rural, Remote and Metropolitan Areas of Australia
Haws	2007	Impact of packaged interventions on neonatal health: a review of the evidence
Hodgins	2016	A New Look at Care in Pregnancy: Simple, Effective Interventions for Neglected Populations
Holtkamp	2017	Factors for successful implementation of population-based expanded carrier screening: learning from existing initiatives
Houweling	2019	The equity impact of community women's groups to reduce neonatal mortality: a meta-analysis of four cluster randomized trials
Hutchinson-Colas	2022	Pregnant Behind Bars
Jackson	2011	Deafness and antenatal care: Understanding issues with access
Jin	2020	Systematic Review of Quality Improvement Processes, Interventions and Structure in Surgery, Obstetric, Trauma, and Anesthesia Systems in Low- and Middle-Income Countries
Khatri	2018	Social determinants of health affecting utilisation of routine maternity services in Nepal: a narrative review of the evidence
Knapp	2006	Economic barriers to better mental health practice and policy
Krumeich	2014	Health in global context; beyond the social determinants of health?
Lisa	2021	Links between biodiversity and human infectious and non-communicable diseases: A review
Little	2019	Cleft Birth Defects and Health Disparities
Lu	2003	Racial and ethnic disparities in birth outcomes: a life-course perspective
Massie	2014	Prenatal and preconception population carrier screening for cystic fibrosis in Australia: Where are we up to?
McCarthy	2019	Pregnancy outcomes for women with pre-pregnancy diabetes mellitus in Australian populations, rural and metropolitan: A review
McKinnon	2016	Do Socioeconomic Inequalities in Neonatal Mortality Reflect Inequalities in Coverage of Maternal Health Services? Evidence from 48 Low- and Middle-Income Countries.
Mingyang	2019	Re: Global inequities in dietary calcium intake during pregnancy: a systematic review and meta-analysis
Moncrieff	2018	The cyclical and intergenerational effects of perinatal domestic abuse and mental health
Moore	2021	Policy Opportunities To Improve Prevention, Diagnosis, And Treatment Of Perinatal Mental Health Conditions
Morgan	2017	Thinking about the environment and theorising change: how could Life History Strategy Theory inform mHealth interventions in low- and middle-income countries?
Mottola	2018	2019 Canadian guideline for physical activity throughout pregnancy
Murphy	2008	Preparation is everything

Nahar	2019	Cultural roadblocks on women's reproductive health rights: a qualitative interpretive meta-synthesis from South Asia and Sub-Saharan Africa
Nicholas	2021	Review of international clinical guidelines related to prenatal screening during monochorionic pregnancies
O'Leary	2016	Screening for Down syndrome in the second trimester of pregnancy
Ochieng	2013	Black African migrants: the barriers with accessing and utilizing health promotion services in the UK
Ogunwole	2020	Community-Based Doulas and COVID-19: Addressing Structural and Institutional Barriers to Maternal Health Equity
Oliveira-Cruz	2003	Approaches to overcoming constraints to effective health service delivery: a review of the evidence
Pedersen	2012	Immigrant women and maternal mortality in Western Europe: A meta-analysis of observational studies
Petticrew	2009	Better evidence about wicked issues in tackling health inequities
Phonyiam	2021	Racial and Ethnic Disparities in Health Care and Health Outcomes for Pregnant Women With Diabetes
Pierle	2019	Genetic Service Delivery Models: Exploring approaches to care for families with hereditary cancer risk
Polacko	2021	Causes and Consequences of Income Inequality – An Overview
Quinn	2014	Provision of maternity care for women in remote Far West New South Wales: How far have we come?
Reading	2014	Global maternal, newborn and child health - so near and yet so far
Requejo	2013	Born Too Soon: Care during pregnancy and childbirth to reduce preterm deliveries and improve health outcomes of the preterm baby
Rezaee	2017	Does Spousal Support Can Increase the Women’s Physical Activity?
Rumbold	2008	A review of the impact of antenatal care for Australian Indigenous women and attempts to strengthen these services
Sanneving	2013	Inequity in India: the case of maternal and reproductive health
Schmied	2010	The nature and impact of collaboration and integrated service delivery for pregnant women, children and families
Schmied	2013	Maternal mental health in Australia and New Zealand: A review of longitudinal studies
Kuhlmann	2016	The importance of community mobilization in interventions to improve sexual, reproductive, and maternal health outcomes: A review of the evidence
Steel	2015	Caesarean section by maternal request
Svanberg	1998	Attachment, resilience and prevention
Tarasoff	2015	Experiences of Women With Physical Disabilities During the Perinatal Period: A Review of the Literature and Recommendations to Improve Care
Taylor	2021	The Importance of Respectful Maternity Care for Women of Color
Tomasi	2004	Health information technology in primary health care in developing countries: a literature review
Wickremsinhe	2019	Beyond "Vessels and Vectors": A Global Review of Registered HIV-Related Clinical Trials with Pregnant Women
Wright	2015	Reproductive Health in the United States: A Review of the Recent Social Work Literature
Yuan	2013	Disadvantaged populations in maternal health in China who and why?
Zwi	2017	The impact of health perceptions and beliefs on access to care for migrants and refugees
Kildea	2019	Reducing preterm births amongst Aboriginal and Torres Strait Islander babies with targeted interventions including increasing the indigenous workforce and providing continuity of midwifery care
N/A	2012	Physical Health

N/A	2012	Populations, Community, and Service Systems
N/A	2012	Quality of Life
Bohren	2018	Methodological development of tools to measure how women are treated during facility-based childbirth in four countries: labor observation and community survey
Crowe	2022	Factors contributing to maternal health inequalities for women who are not white British in the UK
**Reason for Exclusion: No mention of "equit*" or "inequit*"**		
**First Author Last Name**	**Year of Publication**	**Article Title**
Adane	2019	The impact of pre-pregnancy body mass index and gestational weight gain on placental abruption risk: a systematic review and meta-analysis
Adane	2021	The impact of maternal prenatal mental health disorders on stillbirth and infant mortality: a systematic review and meta-analysis
Arab	2018	Healthcare services for Syrian refugees in Jordan: a systematic review
Carey	2018	What principles should guide visiting primary health care services in rural and remote communities? Lessons from a systematic review
Claire	2020	Pharmacological interventions for promoting smoking cessation during pregnancy
Connolly	2021	Mental health interventions by lay counsellors: a systematic review and meta-analysis
de Jong	2017	A systematic literature review on the use and outcomes of maternal and child healthcare services by undocumented migrants in Europe
Fisher	2012	Prevalence and determinants of common perinatal mental disorders in women in low- and lower-middle-income countries: a systematic review
Grigoriadis	2014	Prenatal exposure to antidepressants and persistent pulmonary hypertension of the newborn: systematic review and meta-analysis
Jacques	2019	Prenatal and postnatal maternal depression and infant hospitalization and mortality in the first year of life: A systematic review and meta-analysis
Kingston	2014	Prenatal and Postnatal Maternal Mental Health and School-Age Child Development: A Systematic Review
Lo	2014	Estimating the burden of neural tube defects in low- and middle-income countries
Matin	2021	Barriers in access to healthcare for women with disabilities: a systematic review in qualitative studies
McGeough	2020	Barriers and facilitators perceived by women while homeless and pregnant in accessing antenatal and or postnatal healthcare: A qualitative evidence synthesis
Miah	2013	Does transitional care improve neonatal and maternal health outcomes? A systematic review
Nguyen	2019	Maternal Healthcare Experiences of and Challenges for Women with Physical Disabilities in Low and Middle-Income Countries: A Review of Qualitative Evidence
Norredam	2010	Migrants' utilization of somatic healthcare services in Europe--a systematic review
Ross	2011	Risk for postpartum depression associated with assisted reproductive technologies and multiple births: a systematic review

Shirzad	2021	Prevalence of and reasons for women's, family members', and health professionals' preferences for cesarean section in Iran: a mixed-methods systematic review
Vang	2017	Are immigrants healthier than native-born Canadians? A systematic review of the healthy immigrant effect in Canada
Benkert	2019	Ubiquitous Yet Unclear: A Systematic Review of Medical Mistrust
Khan	2021	Ethnic health inequalities in the UK's maternity services: a systematic literature review
**Reason for Exclusion: Not an interaction with the healthcare system**		
**First Author Last Name**	**Year of Publication**	**Article Title**
Bollini	2009	Pregnancy outcome of migrant women and integration policy: A systematic review of the international literature
Carvalho	2021	A Systematic Policy Review of Black Maternal Health-Related Policies Proposed Federally and in Massachusetts: 2010-2020
Harris-Fry	2017	Determinants of intra-household food allocation between adults in South Asia - a systematic review
Huertas-Zurriaga	2021	Motherhood and decision-making among women living with HIV in developed countries: a systematic review with qualitative research synthesis
O'Brien	2018	The influence of socioeconomic status on gestational weight gain: a systematic review
Saweri	2021	Economic evaluation of point-of-care testing and treatment for sexually transmitted and genital infections in pregnancy in low- and middle-income countries: A systematic review
Vizheh	2021	Women empowerment in reproductive health: a systematic review of measurement properties
Lunze	2015	Innovative approaches for improving maternal and newborn health--A landscape analysis
Miller	2016	Beyond too little, too late and too much, too soon: a pathway towards evidence-based, respectful maternity care worldwide
Gil-González	2006	Knowledge gaps in scientific literature on maternal mortality: a systematic review
Heitkamp	2021	Maternal mortality: near-miss events in middle-income countries, a systematic review
Mac-Seing	2020	Pro-equity legislation, health policy and utilisation of sexual and reproductive health services by vulnerable populations in sub-Saharan Africa: a systematic review
Oliveira-Cruz	2003	Approaches to overcoming constraints to effective health service delivery: a review of the evidence
Chmielewska	2021	Effects of the COVID-19 pandemic on maternal and perinatal outcomes: a systematic review and meta-analysis
Demetrio	2020	Food insecurity in pregnant women is associated with social determinants and nutritional outcomes: a systematic review and meta-analysis
Lassi	2016	Systematic review on human resources for health interventions to improve maternal health outcomes: evidence from low- and middle-income countries
VanDenBerg	2017	Patient-centered early pregnancy care: a systematic review of quantitative and qualitative studies on the perspectives of women and their partners
**Reason for Exclusion: Does not report how equity/inequity influences prenatal care/experience**		
**First Author Last Name**	**Year of Publication**	**Article Title**

Adegbosin	2019	Systematic review and meta-analysis of the association between dimensions of inequality and a selection of indicators of Reproductive, Maternal, Newborn and Child Health (RMNCH)
Amoo	2019	Are there traditional practices that affect men's reproductive health in sub-Saharan Africa? A systematic review and meta-analysis approach
Asciutto	2020	A systematic review of economic evaluations of neonatal and maternal healthcare in immigrant and ethnic minority women
Attree	2005	Low-income mothers, nutrition and health: a systematic review of qualitative evidence
Bhat	2021	Ethnic variation in causes of stillbirth in high income countries: A systematic review and meta-analysis
Batchelor	2021	A Systematic Review of Child Health and Developmental Outcomes Associated with Low Birthweight and/or Small for Gestational Age in Indigenous Children from Australia, Canada and New Zealand
Correia	2021	Telemedicine to deliver diabetes care in low- and middle-income countries: a systematic review and meta-analysis
Cyril	2015	Exploring the role of community engagement in improving the health of disadvantaged populations: a systematic review
Engström	2001	Is general practice effective? A systematic literature review
Gebremedhin	2022	Evaluations of effective coverage of maternal and child health services: A systematic review
Lazzerini	2018	Effectiveness of the facility-based maternal near-miss case reviews in improving maternal and newborn quality of care in low-income and middle-income countries: a systematic review
Long	2018	Prevalence of and reasons for women's, family members', and health professionals' preferences for cesarean section in China: A mixed-methods systematic review
Rahman	2013	Interventions for common perinatal mental disorders in women in low- and middle-income countries: a systematic review and meta-analysis
Saad	2021	Mobile interventions targeting common mental disorders among pregnant and postpartum women: An equity-focused
Shakibazadeh	2017	Respectful care during childbirth in health facilities globally: a qualitative evidence synthesis
Smylie	2010	A review of Aboriginal infant mortality rates in Canada: striking and persistent Aboriginal/non-Aboriginal inequities
Verhoeven	2007	The contribution of teleconsultation and videoconferencing to diabetes care: A systematic literature review
Maness	2016	Associations Between Social Determinants of Health and Pregnancy Among Young People: A Systematic Review of Research Published During the Past 25 Years
Mengesha	2020	Disrespect and Abuse during Childbirth in Ethiopia: A Systematic Review
Lazzerini	2020	Antenatal screening practices in the WHO European Region: a mixed methods study
Kozuki	2013	The associations of birth intervals with small-for-gestational-age, preterm, and neonatal and infant mortality: a meta-analysis
Fiorati	2018	Social iniquities and intersectoriality: Challenge to primary health care
Lagarde	2009	The impact of conditional cash transfers on health outcomes and use of health services in low and middle income countries
Djatmika	2021	Caesarean section as an informed choice in the UK: a systematic review

Gopalan	2017	Maternal and neonatal service usage and determinants in fragile and conflict-affected situations: a systematic review of Asia and the Middle-East.
Goudet	2019	Nutritional interventions for preventing stuning in children (birth to 59 months) living in urban slums in low- and middle-income countries (LMIC) (Review)
Hata	2020	A Systematic Review of Racial and Ethnic Disparities in Maternal Health Outcomes among Asians/Pacific Islanders
Henderson	2021	Aspirin Use to Prevent Preeclampsia and Related Morbidity and Mortality Updated Evidence Report and Systematic Review for the US Preventive Services Task Force
Kim	2013	The Social Determinants of Infant Mortality and Birth Outcomes in Western Developed Nations: A Cross-Country Systematic Review
Kolahdooz	2015	Understanding the social determinants of health among Indigenous Canadians: priorities for health promotion policies and actions
Lassi	2013	Quality of care provided by mid-level health workers: a systematic review and meta-analysis
Mandal	2017	A review of measures of women's empowerment and related gender constructs in family planning and maternal health program evaluations in low- and middle-income countries
Odendaal	2018	Contracting out to improve the use of clinical health services and health outcomes in low- and middle-income countries
Pedersen	2014	Maternal Mortality Among Migrants in Western Europe: A Meta-Analysis
Pedersen	2021	Interventions to reduce preterm birth in pregnant women with psychosocial vulnerability factors–A systematic review
Simonovich	2021	Meta-Analysis Of Antenatal Depression And Adverse Birth Outcomes In US Populations, 2010–20
